# Electrically Robust Single‐Crystalline WTe_2_ Nanobelts for Nanoscale Electrical Interconnects

**DOI:** 10.1002/advs.201801370

**Published:** 2018-12-12

**Authors:** Seunguk Song, Se‐Yang Kim, Jinsung Kwak, Yongsu Jo, Jung Hwa Kim, Jong Hwa Lee, Jae‐Ung Lee, Jong Uk Kim, Hyung Duk Yun, Yeoseon Sim, Jaewon Wang, Do Hee Lee, Shi‐Hyun Seok, Tae‐il Kim, Hyeonsik Cheong, Zonghoon Lee, Soon‐Yong Kwon

**Affiliations:** ^1^ School of Materials Science and Engineering & Low‐Dimensional Carbon Materials Center Ulsan National Institute of Science and Technology (UNIST) Ulsan 44919 Republic of Korea; ^2^ Department of Physics Sogang University Seoul 04107 Republic of Korea; ^3^ School of Chemical Engineering Sungkyunkwan University (SKKU) Suwon 16419 Republic of Korea

**Keywords:** bottom‐up process, electrical performance and reliability, future nanoelectronics, nanoscale interconnect, tungsten ditelluride (WTe_2_)

## Abstract

As the elements of integrated circuits are downsized to the nanoscale, the current Cu‐based interconnects are facing limitations due to increased resistivity and decreased current‐carrying capacity because of scaling. Here, the bottom‐up synthesis of single‐crystalline WTe_2_ nanobelts and low‐ and high‐field electrical characterization of nanoscale interconnect test structures in various ambient conditions are reported. Unlike exfoliated flakes obtained by the top‐down approach, the bottom‐up growth mode of WTe_2_ nanobelts allows systemic characterization of the electrical properties of WTe_2_ single crystals as a function of channel dimensions. Using a 1D heat transport model and a power law, it is determined that the breakdown of WTe_2_ devices under vacuum and with AlO*_x_* capping layer follows an ideal pattern for Joule heating, far from edge scattering. High‐field electrical measurements and self‐heating modeling demonstrate that the WTe_2_ nanobelts have a breakdown current density approaching ≈100 MA cm^−2^, remarkably higher than those of conventional metals and other transition‐metal chalcogenides, and sustain the highest electrical power per channel length (≈16.4 W cm^−1^) among the interconnect candidates. The results suggest superior robustness of WTe_2_ against high‐bias sweep and its possible applicability in future nanoelectronics.

## 1 Introduction

Scaling of integrated circuits (ICs) has led to improved performance and reduced cost of electronic nanodevices, though revolutionary process and materials changes are required to enable technological advances.^1^ Nowadays, circuit performance is no longer limited by the transistors, and the problem of interconnect performance degradation is expected to become more significant. As feature sizes continue to shrink toward the nanoscale, surface and grain boundary (GB) scatterings severely hinder electronic conductivity (a major roadblock to Moore's law at the most fundamental level) owing to the strongly nonlinear increase in their resistivity with scaling.^1^, ^2^, ^3^ The microstructure in nanodevices is no longer stable over practical lifetimes,^2^, ^4^ although the current density should be increased to outperform bulk devices. According to the International Technology Roadmap for Semiconductors,^5^ typical interconnect metals (e.g., Cu and Al) are required in complementary metal oxide semiconductor (CMOS) technology to increase the maximum current density up to ≈5.35 MA cm^−2^ within the cross‐sectional area of 10.8 nm^2^ by 2028. However, it is very difficult for these polycrystalline metals to support such a high current density at the nanoscale because electrical stress‐induced Joule heating and defect‐mediated electromigration cause device degradation^2^, ^3^, ^4^, ^5^, ^6^ in addition to the abovementioned size effects. With a long‐term vision, the most promising prospects for establishing new electrical interconnect technologies that ensure continued validity of Moore's law may be realized by developing fundamentally new interconnect fabrication strategies that gradually replace the prevailing top‐down approach to multilevel metallization via lithography with bottom‐up protocols based on the nanoscale self‐assembly of new interconnect nanomaterials.^1^

Recently, transition‐metal (TM) ditellurides with a distorted 1T‐structure (1T'‐phase), such as WTe_2_ 7, 8, 9, 10 and MoTe_2_,^10^, ^11^ have attracted tremendous interest because of their exotic electronic band structure^7^, ^12^ activating extraordinary electromagnetic characters. For example, they can exhibit nonsaturating, extremely large, and positive magnetoresistance (MR),^8^, ^13^ and can possess type‐II Weyl fermions that evolve topologically protected states.^8^, ^13^ Out‐of‐plane van der Waals (vdW) bonds allow the TM ditellurides to be thinned down to the nanoscale, which triggers new physical phenomena like chiral anomaly induced negative MR^8^ and the quantum spin Hall effect with bandgap opening.^9^, ^11^ Moreover, it is compatible for TM ditellurides to fabricate low‐dimensional metal‐semiconductor heterostructures or local interconnects using their high current‐carrying capacity^14^ and low Schottky barrier to 2D semiconductors,^15^ which promises future nanosized spintronics, electronics, and photonics applications. However, most of the observed novel physical phenomena in TM ditellurides have been demonstrated in mechanically exfoliated samples,^7^, ^8^, ^11^, ^12^, ^14^, ^15^ which are irregular and not scalable. Furthermore, owing to the lack of reliable production methods for the materials, high‐field transport studies of type‐II Weyl semimetal systems under different ambient conditions are rare compared to the studies of Dirac semimetals (e.g., graphene) at the present stage, even though large current‐carrying capacities are expected based on their fast carrier mobility.^16^ On top of that, a systematic investigation of the influence of dimensions on the electrical breakdown through TM ditellurides or even other metallic TM chalcogenides (e.g., TiS_2_, TaS_3_, NbSe_3_, and ZrTe_3_) has not yet been carried out, though this would enable us to understand the fundamental physics of how the interface area impacts carrier transport and scattering mechanisms. Therefore, for practical device applications, it is important to achieve the bottom‐up synthesis of the single‐crystalline TM ditellurides with high electrical performance and reliability under current‐carrying conditions by understanding the electrical breakdown mechanism.

Here, we report low‐ and high‐field electrical properties of quasi‐1D‐like WTe_2_ single crystals, mainly focusing on their electrical breakdown and current‐carrying capacities under various ambient conditions. The bottom‐up growth mode of WTe_2_ nanobelts allowed us to systemically characterize the electrical properties of single‐crystalline WTe_2_ crystals as a function of channel dimensions (i.e., cross‐sectional area) by preventing random crystalline orientation and degraded edges (by harsh etching) of the crystals (which is unavoidable for the exfoliated flakes). The breakdown of single‐crystalline WTe_2_ devices under vacuum and with AlO*_x_* capping layer was found to follow the ideal behavior for Joule heating and especially the AlO*_x_*‐capped WTe_2_ nanobelts (a practical interconnect design, where interconnects are encapsulated by dielectrics^1^) endured large input power in spite of their high resistivity. The grown WTe_2_ nanobelts exhibited a remarkably high breakdown current densities (*J*_B_) of up to ≈100 MA cm^−2^ (almost twice greater than the previously reported value for exfoliated flakes^14^), which are remarkably higher than conventional metals and other vdW atomic crystals. Furthermore, WTe_2_ nanobelts could sustain the highest electrical power per channel length of ≈16.4 W cm^−1^ among the interconnect candidates, even higher than reported graphene films prepared by top‐down and bottom‐up processes.

## 2 Results and Discussion

Chemical vapor deposition (CVD) has so far adopted for the growth of layered vdW atomic crystals on various substrates;^10^, ^17^ however, the production of high‐quality, stoichiometric WTe_2_ layers using CVD process remains an unsolved challenge due to the low stability and activity of Te, and difficulties in Te incorporation during growth.[[qv: 10,17b]] Inherently, the synthetic films produced are polycrystalline and therefore the GBs are expected to degrade the intrinsic properties of the resulting films.[qv: 10,17] These problems prevent studying the intrinsic electrical properties and carrier‐carrying capacity of WTe_2_ especially on a nanoscale, owing to electron scattering and disorder effects.^18^, ^19^ Very recently, we established a strategy to introduce Cu*_x_*Te*_y_* (or Au*_x_*Te*_y_*) eutectic alloy to overcome these problems, providing a liquid‐like Te‐rich ambient condition adequate to make stoichiometric binary and ternary single crystals composed of TM ditellurides.^20^ We note that any salt promoters (e.g., NaCl or KCl),^21^ which have recently been used for 2D growth of TM chalcogenides, have not been considered in this work to prevent the inclusion of any unintentional impurities and to avoid the need for additional processes to define the device channel.

**Figure 1:**
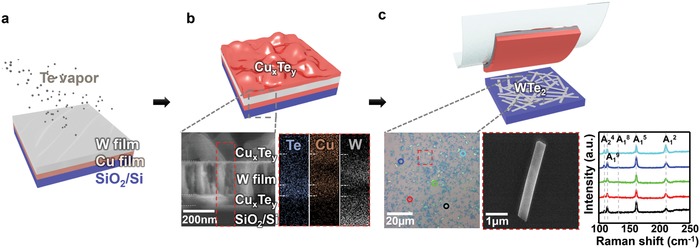
WTe_2_ nanobelts directly prepared on a SiO_2_/Si substrate. a–c) Experimental scheme used to prepare WTe_2_ nanobelts on SiO_2_/Si. Generally, synthesis is conducted at *T* = 500 °C by tellurization of predeposited W/Cu layers on the substrate as schemed in (a). As‐reacted eutectic matrix of Cu*_x_*Te*_y_* illustrated in (b) enables growth of WTe_2_ within itself. Inset of (b) shows (left) cross‐sectional SEM image of as‐reacted sample displaying the composed material (Cu*_x_*_′_Te*_y_*_′_/W/Cu*_x_*Te*_y_*), and (right) corresponding EDS mapping image. (c) A schematic showing as‐prepared WTe_2_ exposed on the substrate after “tape‐treatment,” which denotes removal of the by‐products of Cu*_x_*Te*_y_* and unreacted W layers by peeling off using adhesive tape. Inset of (c) shows (left) OM and (middle) SEM images of tape‐treated WTe_2_ nanobelts on SiO_2_/Si, grown for *t* = 2 min, and (right) Raman spectra of WTe_2_ nanobelts characterized by using 514.5 nm laser as a light source. Raman spectra from each point marked in a left OM image show five distinct vibrational modes of WTe_2_, indicating uniform distributions of WTe_2_ in the tape‐treated sample.

**Figure** **1** illustrates a schematic for WTe_2_ growth on SiO_2_/Si that relies on a eutectic alloy‐assisted synthesis approach.^20^ Before the growth, a W–Cu–SiO_2_/Si sample was located ≈1 cm above the Te‐powder inside a furnace system (Figure S1a,b, Supporting Information). Note that, to investigate the degree of degradation of the as‐synthesized WTe_2_, the reverse sequence of a W–Cu–SiO_2_/Si sample (this study) is formed by reversing the deposition steps of W and Cu on SiO_2_/Si (i.e., Cu–W–SiO_2_/Si) (ref. 20). In this study, vaporized Te reacts with Cu at growth temperature *T*, yielding a eutectic matrix of Cu*_x_*Te*_y_* located above/below the W layer (Figure S1c, Supporting Information) and the liquid‐like Cu*_x_*Te*_y_* formed just below the W layer (i.e., above the SiO_2_/Si substrate) accelerates the formation of WTe_2_ (details of the growth mechanism are presented in Note S1 and Figure S2 of the Supporting Information). After cooling to room temperature, high‐density WTe_2_ crystals were prepared directly on SiO_2_/Si by “tape‐treatment” (Figure S3, Supporting Information), a method to remove the as‐reacted by‐products without any chemical etching followed by a H_2_ bubbling transfer process based on water electrolysis.^20^ In this way, the degradation of WTe_2_ single crystals after growth could be minimized (Figure S4, Supporting Information), achieving a significant increase in electrical properties.

The as‐synthesized WTe_2_ crystals show a belt‐like anisotropic 1D morphology with flat surface and uniform thickness (Figure 1c; Figure S5 (Supporting Information)) by combining scanning electron microscopy (SEM) and atomic force microscopy (AFM). Raman spectra and X‐ray diffraction (XRD) of the nanobelts (Figure 1c; Figure S6a (Supporting Information), respectively) exhibit all expected peak positions for WTe_2_ 10, 22 (without any observable change in Raman signals in Figure S7 (Supporting Information)). We can easily recognize the longest direction of the nanobelts using optical microscopy (OM) from polarized Raman analysis^20^ (Figure S8, Supporting Information). High‐resolution transmission electron microscopy (HR‐TEM) images reveal that the WTe_2_ nanobelts have the 1T'‐phase with an elongated shape along the *a*‐axis (Figure S9a, Supporting Information). The measured unit cell size is significantly close to the computationally obtained one (Figure S9b,c, Supporting Information). Uniform atomic distributions of W and Te without noticeable defects are observed across the nanobelts in the scanning TEM (STEM) image as well as in the TEM‐EDS mapping (Figure S9d–e, Supporting Information), further confirming the high quality of the crystal. The high stoichiometry of the WTe_2_ and complete removal of by‐products after tape‐treatment were verified by XRD and SEM‐EDS (Figure S6, Supporting Information).

From growth‐parameters‐dependent study, we observed the significantly different growth rate of WTe_2_ crystals in the Cu*_x_*Te*_y_*(*l*) droplets depending on the crystallographic directions (Figure S5e, Supporting Information). We note that a significant dimensional control of WTe_2_ crystals was possible by decreasing *T* and *t* for potential application in future nanoelectronics; e.g., the cross‐sectional area of WTe_2_ nanobelts could be reduced to *W* = 59.8 ± 29.4 nm and *H* = 3.7 ± 1.7 nm for the samples grown at *T* = 450 °C and *t* = 0 min (Figure S5f, Supporting Information). In principle, there is more room to narrow down the average size and standard error of crystals grown while decreasing *T* once it is higher than 340 °C, as discussed in Note S1 (Supporting Information).

**Figure 2:**
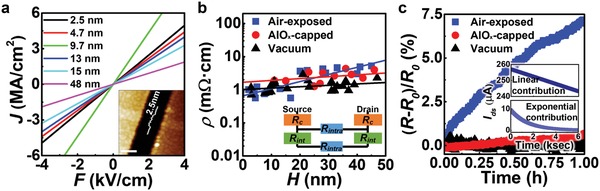
Low‐bias electrical properties of WTe_2_ nanobelts under various ambient conditions. a) Representative current density depending on electric field (*J–F*) curve indicating the well‐defined Ohmic contact shown as linear behavior. Inset shows a representative AFM image of an electrical device made from the thinnest WTe_2_ channel we tested (along the *a*‐axis) with two‐terminal Au/Ti contacts, showing the channel thickness of a device ≈2.5 nm. The scale bar in the AFM image is 400 nm. b,c) Resistant behavior change of WTe_2_ interconnects with ambient differences at *V*_ds_ = 0.3 V. b) Plot showing resistivity (ρ) of WTe_2_ nanobelts with different channel thickness (*H*). The positive slop of the *H*–ρ graph (d*ρ/*d*H ≈* 4.2 µΩ cm nm^−1^ for samples under vacuum, and 21.5 µΩ cm nm^−1^ for air‐exposed ones) might be attributable to the existence of interlayer resistances (*R*_int_). Inset of (b) represents resistor network model that explains increase in ρ as a function of the number of layers. c) In situ resistance change (Δ*R/R*_0_) of samples under varied ambient, depending on sampling time. Insets show current (*I*_ds_) decreases in the air‐exposed WTe_2_ device as a function of measurement time, plotted by fitted parameters of linear (above) and exponential (below) contributions.

**Figure** **2**a presents typical current density (*J*) versus electrical field (*F*) characteristics of the two‐probe WTe_2_ devices (along the *a*‐axis) with different channel thicknesses (*H*) under vacuum. One can easily find that the channel has a uniform width and thickness from an AFM image (inset in Figure 2a). The perfectly linear *J*–*F*s at low electric fields indicate a well‐defined Ohmic contact after the ultrahigh vacuum (UHV) annealing process (Figure S10a, Supporting Information), regardless of the investigated channel thickness (2 < *H* < 50 nm). We note that the WTe_2_ devices sustained high *J* of 4.07 ± 0.84 MA cm^−2^ at low *F* of 4 kV cm^−1^, which are comparable to those for exfoliated flakes,^14^ showing the high quality of bottom‐up grown WTe_2_ nanobelts.

Figure 2b shows the changes in the resistivity (ρ) of the WTe_2_ devices at a low bias (*V*_ds_ = 0.3 V) depending on the ambients and *H*, without any subtraction of contact resistance (*R*_c_) effect. The devices under vacuum had a low resistivity of 1.62 ± 0.85 mΩ cm, slightly lower than that of exfoliated flakes.^14^, ^23^ The temperature‐dependent electrical characteristic of ρ further indicates the crystals' high quality as well as their intrinsic metallic behavior (Figure S11, Supporting Information). In contrast to the WTe_2_ under vacuum, the air‐exposed samples had a higher resistivity of 3.09 ± 1.5 mΩ cm that might be caused by channel degradation, because WTe_2_ is vulnerable to air‐ and moisture‐induced oxidation^18^, ^19^ (Figure S10b, Supporting Information). We employed the 3 nm thick AlO*_x_*‐capping layer for the WTe_2_ devices to avoid air degradation; however, the measured resistivity of AlO*_x_*‐capped samples was 3.54 ± 2.35 mΩ cm. We believe that an avoidable air‐exposure (<15 min) during sample preparation for the atomic layer deposition (ALD) process or high *R*_C_ might lead their higher resistivity (Here, we estimated *R*_c_ using both the two‐probe model (Figure S12, Supporting Information) and transfer‐length method (Figure S13, Supporting Information) and compared them in Figure S10c (Supporting Information). The *R*_c_‐excluded ρ as a function of *H* was plotted in Figure S10d (Supporting Information)). Nevertheless, we find that air‐passivation is critical for WTe_2_ to sustain its intrinsic electrical properties. We observed upsurges in the resistance of an air‐exposed device with time because of channel degradation process, contrary to those under vacuum and with AlO*_x_* capping layer (Figure 2c; Figure S14 (Supporting Information)). From the electrical data fitting (see Note S2 of the Supporting Information for more details), we found that the permanent degradation of current was quite fast for tested WTe_2_ nanobelts (≈34 A cm^−2^ s^−1^) (higher than Cu^24^ (≈0.62 nA cm^−2^ s^−1^) and phosphorene^25^ (≈0.55 mA cm^−2^ s^−1^), but lower than MXene^26^ (≈1.8 kA cm^−2^ s^−1^)), implying the importance of air‐passivation for WTe_2_.

**Figure 3:**
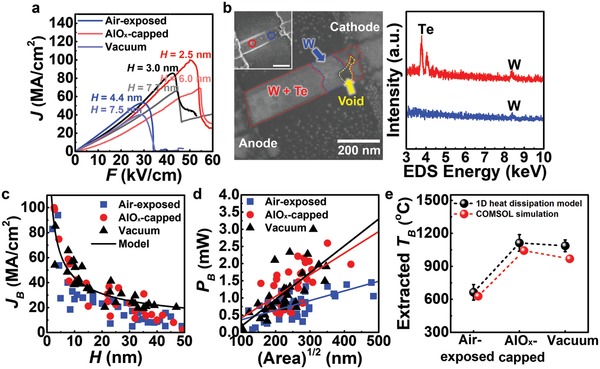
Electrical breakdown of WTe_2_ devices during high electrical stress. a) Representative current density (*J*) versus electrical field (*F*) features of WTe_2_ interconnects showing abrupt decreases in *J*. b) SEM‐EDS analysis of a device broken by electrical failure. (left) Representative SEM image with the BSE mode of the WTe_2_ interconnect, noted with the atomic distributions after the failure. Inset of (b) is the corresponding SEM image in the SE mode wherein the formed void (blue circle) is significantly different from the pristine one (red circle). (Right) EDS spectra of marked red and blue points at the SEM image, showing the Te deficiency at the void. c) Breakdown current density (*J*_B_) of the measured WTe_2_ devices versus channel thickness (*H*) in various ambient conditions. A solid line indicates a curve fitted to the 1D heat dissipation model (i.e., *J*_B_ ∝ *H* ^−1/2^). d) Input power (*P*_B_) into WTe_2_ channel shortly before electrical failure, as a function of each channel's contacted area ((*WL*)^1/2^) to substrate. e) Averaged maximum temperature (*T*_B_) extracted in accordance with pure Joule heating mechanism (black) and by finite‐element simulation performed using a COMSOL modeling software (red).

We now shift our focus to the high‐field electrical properties. We conducted electrical field sweep up to failure of the WTe_2_ devices and the representative *J* versus *F* plot is presented in **Figure** **3**a. The current‐carrying capacity of the WTe_2_ devices increases to a breakdown current density (*J*_B_) and then decreases abruptly. We found that the ρ (reciprocal slope of *J*–*F* graph) slightly lowers as the current increases. This tendency is analogous to a trend of reducing ρ at an increased ambient temperature (Figure S11a, Supporting Information), which is the first evidence of Joule heating until the electrical breakdown. As current flows significantly, electrically induced Joule heating typically stresses devices, resulting in increases in temperature and finally failure by atomic displacements.

The formation of nanosized voids along the channel after high‐field sweep shows that the failure happened along the WTe_2_ nanobelts, not within the electrical contacts, as marked with a blue circle to stand out against the pristine one (red circle) in the inset of Figure 3b. By comparing the SEM images with the secondary electron (SE) and back‐scattered electron (BSE)[[qv: 17a]] modes and the EDS spectra, we could precisely distinguish the atomic distribution of W and Te after the electrical failure, as shown in Figure 3b. Because the heavier metal, W, is displayed brighter in the BSE mode, the darkest region (marked by a yellow arrow) is where both W and Te do not exist; thus, we assume that the first atomic displacement occurred in this region. The area that consists of only W and not Te (marked by a blue arrow) demonstrates that the electrical breakdown severely affects the atomic movement of the Te atoms. Considering easy evaporation of Te (evaporation of Te power begins at ≈400 °C under vacuum), the unstable and displaced Te atoms possibly disappeared due to the elevated temperature of the self‐heated WTe_2_ channel (the weakness of Te under electrical stress is further discussed in the note for Figure S15, Supporting Information).

High *J*_B_ over 40 MA cm^−2^ could be observed in the few‐layered devices (*H* < 10 nm), and remarkably, even higher *J*_B_ of *≈*100 MA cm^−2^ was shown for a 2.5 nm thick (three‐layered) device, as summarized in Figure 3c. We found that the devices exhibited thickness‐dependent current‐carrying capacities, similar to the studies on nanowires' *J*_B_, which are reliant on their diameter.^27^, ^28^ We plotted the *J*_B_ as a function of *H*, obeying the following 1D thermal dissipating relation^6^, ^14^, ^29^

(1) $$P_{B}\;\;=\;\;g\left(T_{B}\;\;-\;\;T_{0}\right)L$$

or

(2) $$J_{B}\;\;=\;\;{\left[\frac{g\left(T_{B}\;\;-\;\;T_{0}\right)}{\rho_{B}HW}\right]}^{\frac{1}{2}}\;\propto \;\;H^{-\frac{1}{2}}$$

where *P*_B_ is the power flowing into channel by Joule heating, *g* is the thermal conductance of the channel per unit length to the substrate, *T*_B_ is the Joule heating‐induced maximum temperature at breakdown, *T*_0_ is the ambient temperature, and ρ_B_ is a resistivity shortly before failure. The value of *J*_B_ versus *W* also agrees well with the model (Figure S16, Supporting Information); however, the value of *J*_B_ seems to rely on *H* rather than *W* in the nanobelts.

**Figure 4:**
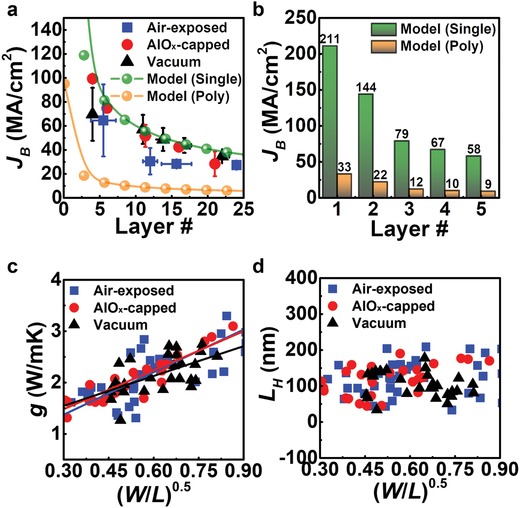
Electrical characteristics of WTe_2_ interconnects during high current injection. a) Comparisons of experimental data and calculated breakdown current density (*J*_B_) as a function of the number of layers. The 1D heat dissipation model and average of the parameters for the thinner WTe_2_ is used to calculate *J*_B_. For the polycrystalline structure (orange circle), thermal conductivity[[qv: 17b]] of *k* = 0.8 W (mK)^−1^ and the Wiedemann–Franz law^30^ *k*/σ ∝ *T* is used to extract electrical conductivity σ. Thickness of WTe_2_ was converted to layer number by considering interlayer distance. b) Ideal *J*_B_ for the single‐ and polycrystalline WTe_2_ dependent on layer number. *J*_B_ up to 221 MA cm^−2^, can be achieved if a negative bandgap is sustained in a few layers. c) Calculated thermal conductance to substrate (*g*) with varied geometry factor ((*W*/*L*)^1/2^). It demonstrates a linear relation between *g* and (*W*/*L*)^1/2^, as shown in the solid lines. d) The calculated characteristic healing length (*L*_H_ = 100 nm on average) for all tested devices. The details on the extract method are included in the Note S3 of the Supporting Information.

The ideal *J*_B_ for WTe_2_ is calculated and demonstrated, using Equation (2) and the parameters. The obtained *J*_B_ is consistent with this calculated trend, as shown in **Figure** **4**a (and also see Figure 3c). We also found that the *J*_B_ of monolayer WTe_2_ may reach ≈211 MA cm^−2^ in theory if it sustains metallic behavior at that thickness (Figure 4b). (As for the monolayer device, we could not obtain any reliable data because degradation of a monolayer of WTe_2_ is quite fast; less than 13 min is required for complete oxidation under air.^19^) However, it has been recognized that bandgap opening due to the quantum spin Hall effect happens in such a thin layer (<three layers),^9^, ^11^ indicating the obtained *J*_B_ (*≈*100 MA cm^−2^, Figure 3a) for the 2.5 nm thick WTe_2_ in this study is very close to the highest value that one can realize. Note that the intrinsic metallic behavior lasted at our few‐layer WTe_2_ with a negligible disorder effect^31^ (Figure S11c, Supporting Information). Moreover, higher *g* causes higher input power (*P*_B_ = *I* ^2^_B_(*R*_B_–*R*_C_)) and higher *J* until the breakdown, which is significantly affected by the channel area (*W × L*) contacted to the substrate. This is because the fast heat transport through the contacted area into SiO_2_/Si substrate contributed to the overall *g* of the channel, as implied in Figure 4c. Note that Equations (1) and (2) do not have any contributions from lateral transport into metal electrodes because our samples' shorter characteristic healing length *L*_H_ (≈100 nm on average (Figure 4d) than the channel length (*L* >> *L*_H_) allows almost all the heat to transport mainly through substrate vertically, as discussed in Note S3 (Supporting Information).

We found that *P*_B_ of devices is dependent on their ambient conditions. The vacuum‐ and AlO*_x_*‐capped samples showed the higher averaged *P*_B_ than those under air ambient, indicated as greater slope in Figure 3d. This indicates that power sustaining ability could be degraded by air, particularly for the larger area channel. To unveil this effect, we extracted *T*_B_ using Equation (2) and the trend of *T*_B_ follows the variation in *P*_B_ expectedly (Figure 3e). Notably, the *T*_B_ of devices under vacuum and with AlO*_x_* capping layer could approach the melting point of the WTe_2_ (*T*_m_ ≈ 1020 °C).^32^ Thus, we can say that the vacuum‐ and AlO*_x_*‐capped samples failed almost purely by the Joule heating mechanism. However, the air‐exposed samples' lower *T*_B_ represents that the other failure mode was activated in tandem with self‐heating. To validate the thermal transfer model, we calculated *T*_B_ for the samples in various ambient conditions by finite‐element simulation, and the results fit well into the model as shown in Figure 3e, and Figure S17 (Supporting Information).

**Figure 5:**
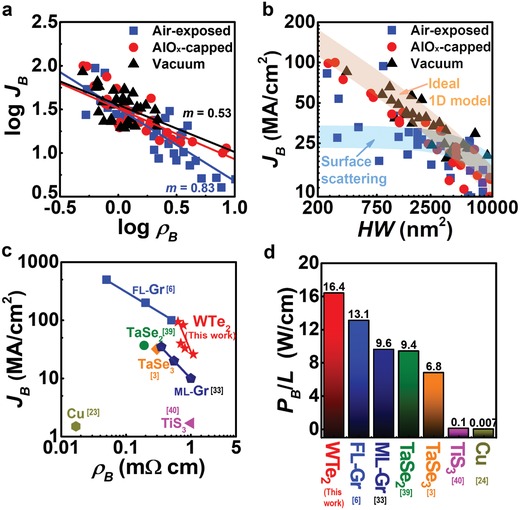
Analysis of electrical breakdown mechanism and comparison of current‐carrying capacity of interconnect candidate materials under air ambient. a) Logarithm *J*_B_–ρ_B_ plot of WTe_2_ interconnects with varied ambient conditions. The extracted *m* using a power law of *J*_B_ = *kρ*_B_^−^ *^m^* for each condition is noted, which is a useful signal to infer the rate of Joule heating until failure. b) *J*_B_ versus cross‐sectional area (*WH*) of the channel shows the size effect by edge scattering on air‐exposed WTe_2_ transport. The calculations using ideal 1D heat dissipation model (orange) and scattering model (light blue) are plotted as solid lines. The parameters used for calculation were *W =* 150–450 nm, *L* = 500 nm, *p* = 0.85, and *l*_0_ = *W*/4. c) Maximum current density until breakdown (*J*_B_) of each material regarding its resistivity. d) Averaged maximum input power per channel length (*P*_B_/*L* (=*J*_B_^2^ρ_B_ *HW*)) for different channel materials that can endure under air.

Next, we tried to analyze electrical breakdown mechanism systematically by means of a power law that is a modified version of Equation (2)

(3) $$J_{B}\;\;\propto \;\;\rho_{B}^{-m}$$

where *m* is 0.5 for ideal Joule heating, and exceeds 0.5 for the defect‐induced electromigration generally, which is a good indicator of the rate of breakdown by self‐heating.^6^, ^33^ **Figure** **5**a shows the *J*_B_ versus ρ_B_ on a logarithmic scale to fit to Equation (3). The lowest *m* of 0.52 for AlO*_x_*‐capped samples indicates the robustness of channel under high‐bias, probably due to further suppression of electromigration (i.e., surface diffusion) by passivation (Figures S15 and S18, Supporting Information). By contrast, the air‐exposed samples possessed the highest extracted *m* value of 0.83, implying that due to the defect‐induced electromigration, the failure occurs faster than the intrinsic stability (the failure occurs even before the temperature reaches *T*_m_).

To further understand the electromigration‐related breakdown mechanism, we investigated the failure locations within the channels depending on the ambients (Figure S15, Supporting Information). In our WTe_2_ devices, we found that the change in the failure point location depends on the isolation of air and prevention of atomic migration on the surface, which vary strongly with ambient conditions and to a lesser extent with sample thickness (*H*) and contact resistance (*R*_c_). For the air‐exposed samples, the damage was observed to be caused by the electrical stress near the cathode, and not at the center or near the anode. Since the electrons bombard the atoms as they move from the cathode to anode under the effect of the electron wind force (*F*_wd_)^1^, ^4^ along the self‐heated WTe_2_ nanobelt, this electromigration could also transport the W or Te atoms especially those at the surface of the WTe_2_ nanobelt, along the same direction. This increases the possibility of defect formation near the cathode, which is observed as voids after all (Figure S15a, Supporting Information). Regarding the electromigration process in atomic scale, we believe that the atomic displacement (including the process of breaking the W—Te bonds) took place first, under the influence of both electron bombardment and self‐heating; and then the unstable Te atoms (rather than the W atoms) possibly evaporated. Compared to air‐exposed samples, the damaged locations of the AlO*_x_*‐capped devices were observed to be closer to the center (Figure S15b, Supporting Information), because the Joule heating induced failure occurs as a result of the increase in temperature: as temperature of the WTe_2_ devices reaches *T*_m_, WTe_2_ cannot continue to exist in its solid form. Hence, a failure could occur at a location regardless of the location of the application of *F*_wd_ with respect to the cathode. This indicates that the AlO*_x_* capping layer has retarded the electromigration, possibly by blocking the absorption of the ambient molecules on the lattice (which give rise to structural vulnerability and vacancy formation) and by physically restraining the atomic migration on the surface (for more details, please see the note for Figure S15, Supporting Information).

The effect of air exposure on current‐carrying capacity was further investigated by surface scattering model as a function of cross‐sectional area of channel (*W × H*). Taking thickness‐dependent resistant behavior into account, we recalculated 1D heat transport model with Fuchs–Sondheimer model^34^, ^35^ for the electron‐nanobelt edge scattering (see Note S4 of the Supporting Information for more details) and this is shown as blue line in Figure 5b. Here, some air‐exposed devices deviated randomly from ideal 1D model (gray line), but they followed the size effect by edge scattering (blue line). Since the oxidation usually starts from edge of nanomaterial, carrier transport along the quasi‐1D metallic bonds could be more diffusive especially at the edge under air ambient. This implies that air‐passivation is required to maintain its high *J*_B_ without deterioration from edge scattering at along narrow channels.

However, we found that none the vacuum‐devices' *J*_B_ showed any evidence of severe surface scattering as (*W × H*) decreases down to ≈200 nm^2^, consistent with calculations using the ideal 1D model (gray line). This is very interesting because nanoribbons prepared by the top‐down process usually show degraded electrical properties for such narrow channels.^2^, ^23^ Contrary to the WTe_2_ flakes prepared by the top‐down processes,^14^, ^23^, ^36^, ^37^ our WTe_2_ nanobelts may show rather scattering‐free carrier motion along the contained metallic dimers. This is possible owing to the absence of any degraded edges by harsh etching^23^, ^36^ and random crystalline orientation^14^, ^37^ in our WTe_2_ nanobelts, that prevent deterioration of current flow by backscattering. The dangling‐bond‐free, smooth vdW surface also contributes to elastic reflection of carriers on the surface.^2^, ^3^, ^34^ Furthermore, the single‐crystalline nature of WTe_2_ helps to avoid GB scattering, which is also an important mechanism to lower conducting capacity. These negligible scattering effects indicate that we can control an increase in resistivity, permitting high current‐carrying capacity. We suppose that carrier transport along low noisy metallic bonds in WTe_2_ also help to provide such a high durability against electrical stress, as recently reported in a quasi‐1D vdW material, TaSe_3_.^38^

Compared to other candidate materials, our WTe_2_ promises great advantages as a local interconnect with respect to *J*_B_ (Figure 5c). The *J*_B_ of WTe_2_ (up to ≈94 MA cm^−2^, under air) in our work is higher than Cu^24^ (several MA cm^−2^), and even more than other vdW nanomaterials, such as TaSe_2_ 39 (≈37 MA cm^−2^), TaSe_3_ 3 (≈32 MA cm^−2^), and TiS_3_ 40 (≈1.7 MA cm^−2^). The obtained value is close to that of graphene (*J*_B_ of several hundred MA cm^−2^ for mechanically exfoliated few‐layered graphene (FL‐Gr)^6^ and ≈40 MA cm^−2^ for CVD‐grown multilayer graphene (ML‐Gr)^33^), which is a well‐known material that can carry larger *J*_B_ than any other vdW materials. The high *J*_B_ in WTe_2_ crystals is peculiar with respect to their higher resistivity (*≈*1 mΩ cm) compared to other vdW materials, indicating their high stiffness against electrical stress.

To further compare the materials' reliability under high‐voltage sweep, we estimate that the maximum electrical power per channel length that a material can tolerate on the brink of breakdown is *P*_B_/*L* (Figure 5d). Importantly, WTe_2_ nanobelts could sustain the highest *P*_B_/*L* of ≈16.4 W cm^−1^ among the interconnect candidates, even higher than those of mechanically exfoliated FL‐Gr^6^ (≈13.1 W cm^−1^) and CVD‐grown ML‐Gr^33^ (≈9.6 W cm^−1^). This shows the superior robustness of WTe_2_ against electrical breakdown, which might be attributed to the high *T*_B_ (≈1001 °C under vacuum, ≈664 °C under air), particularly compared with those of graphene^33^ (≈600 °C) or TiS_3_ 40 (≈450 °C) under oxygen‐rich condition (Figure S19a, Supporting Information). The dimension difference of the channel could induce a change in a capacity for heat transfer as *g* ∝ (*W*/*L*)^1/2^, although the effect was not sufficient to make a significant difference (Figure S19b, Supporting Information). It is worthwhile to note that polycrystalline Cu obviously had a low value of *P*_B_/*L*, despite its high *T*_m_, because the electrical failure happens mainly due to GB migration, not self‐heating. Therefore, the GB‐free nature of WTe_2_ nanobelts also significantly helped to enhance their electrical reliability.

## 3 Conclusion

We have performed low‐ and high‐field electrical characterization of single‐crystalline WTe_2_ nanobelts obtained via a bottom‐up process under various ambients. We found that the breakdown of WTe_2_ devices under vacuum and with AlO*_x_* capping layer follows the ideal behavior for Joule heating, far from edge scattering. As opposed to the WTe_2_ flakes prepared by the top‐down process, our WTe_2_ nanobelts show rather scattering‐free transport behavior along their quasi‐1D atomic intermetallic chains, owing to the absence of any degraded edges by harsh etching and random crystalline orientation, as well as the single‐crystalline nature of WTe_2_ with a dangling‐bond‐free, smooth vdW surface. Accordingly, the WTe_2_ nanobelts could exhibit a higher *J*_B_ of up to ≈100 MA cm^−2^ as compared to conventional interconnect metals and other TM chalcogenides, and sustain the highest *P*_B_/*L* of ≈16.4 W cm^−1^ among the interconnect candidates.

Although direct integration with the CMOS process seems difficult at this stage, we believe that our strategy sufficiently shows the potential for applications in future downscaled nanoelectronics technology in terms of the interesting fundamental electrical properties of quasi‐1D‐like WTe_2_ single crystals compared to other vdW materials. Moreover, our work provides the first direct insights into the role of channel size on the Joule heating‐induced electrical breakdown of layered TM chalcogenides and type‐II Weyl semimetals. We believe that further size reduction of our nanobelts to orders of less than 10 nm by spontaneous growth would facilitate their application for “post‐CMOS interconnect”, which is a requirement because of the limited signaling and reliability caused by interconnect technology with downscaled IC features (we calculated how the performance of WTe_2_ interconnect could be improved in percentage in Note S5 of the Supporting Information). We also suggest that the introduction of doping procedures using intercalation^2^ or substitutional^20^ dopants will provide a practical pathway to increase carrier density and to modulate the work function for WTe_2_ interconnects with lower resistivity and contact resistance. With steady improvements in growth/fabrication techniques and 1D nanomaterial springboards, we expect that our nanobelts will develop into a large field of their own.

## 4 Experimental Section

*Growth of WTe_2_ Nanobelts*: WTe_2_ was grown using a similar setup as that for conventional powder vaporization; the schematic of the setup used in this work is shown in Figure S1, Supporting Information. To prepare metal precursors (predeposited sample of W–Cu–SiO_2_/Si) before growth, the Cu film (50 nm) was deposited onto Si/SiO_2_ by using an UHV e‐beam evaporator with a high‐purity Cu solid source (99.99% Cu pellet). A W layer (20–200 nm) was fabricated on this Cu–SiO_2_/Si sample using a direct current magnetron sputtering system under the optimized deposition conditions (less than ±5% uniformity within the wafer). Next, the prepared W–Cu–SiO_2_/Si was suspended above a quartz boat containing 0.1 g Te powder (Sigma‐Aldrich, 99.5%), which was located at the center of the furnace. By simply heating the reactants at *T* = 450–500 °C and atmospheric pressure using Ar (500 sccm) as the carrier gas in an 8 in. quartz tube during the designated growth time (usually *t* = 0–120 min, depending on the experimental conditions), WTe_2_ crystals inside the Cu*_x_*Te*_y_* matrix located above/below the W layer (Cu*_x_*_′_Te*_y_*_′_/W/Cu*_x_*Te*_y_*/SiO_2_/Si) could be prepared. After the growth, the system was cooled to room temperature by opening the furnace cover. The tellurization for *t* = 0 min (i.e., shoot) implies that the growth furnace was turned off when reaching to the growth temperature *T*.

The WTe_2_ crystals were analyzed after removing Cu*_x_*Te*_y_* by “chemical etching” or “tape‐treatment.” For the “tape‐treatment” method, the tape was first placed on the as‐grown sample. A compressive force was applied manually to ensure good adhesion between the tape and the sample. By simply peeling off the tape from the sample, the Cu*_x_*_′_Te*_y_*_′_/W/Cu*_x_*Te*_y_* structure could be removed from the Si/SiO_2_ substrate, leaving behind WTe_2_. Using this method, most of the other materials except WTe_2_ were removed from the substrate. It is noted that removal of those by‐products like W and Cu compounds can be effectively accomplished using an ammonium persulfate (APS) etchant as well,^20^ but an APS water‐containing solution degrades their performance by oxidation and/or defect formations of WTe_2_ crystals if they are exposed for long time (Note S1 and Figure S4, Supporting Information). Chemical etching of the Cu compounds was performed by dipping the as‐synthesized sample in 1 and 30 m APS solutions for ≈1 h (slow etching) and 2 min (fast etching), respectively. The etched‐sample was rinsed with isopropyl alcohol (IPA) and then with deionized water to remove any APS residue.

*Structural Characterization*: The sample morphology was investigated based on SEM (Hitachi S‐4800) images recorded at an accelerating voltage of 7 kV. EDS analysis was conducted using the SEM setup at 15 kV. For compositional analysis by EDS, the crystals were attempted to transfer onto another substrate in order to avoid overlapping of the W M‐shell and Si K‐shell peaks at ≈1.7 keV. For the transfer, the crystals were dispersed in IPA by sonication and then dropped onto the other desired substrate using a pipette at an elevated temperature (≈90 °C), on a hotplate.

Micro‐Raman measurements were carried out with the 514.5 nm (2.41 eV) line of an Ar ion laser. The laser beam was focused onto a single WTe_2_ nanobelt by using a 50× objective lens (0.8 N.A.). The scattered light was collected by the same objective lens and dispersed with a Jobin‐Yvon Horiba iHR550 spectrometer (2400 grooves mm^−1^) and detected with a liquid‐nitrogen‐cooled back‐illuminated charge‐coupled‐device array detector. Volume holographic filters (Ondax and Optigrate) were used to reject the Rayleigh‐scattered light. The laser power was kept at 100 µW to avoid local heating of the samples. The spectral resolution was below 1 cm^−1^. For polarized Raman measurements, the 632.8 nm (1.96 eV) line of a He‐Ne laser was used. An achromatic half‐wave plate was used to rotate the polarization direction of the linearly polarized incident laser beam to the desired direction. The analyzer angle was set such that photons with polarization parallel to the incident polarization pass through (parallel configuration). Another achromatic half‐wave plate was placed in front of the spectrometer to keep the polarization direction of the signal entering the spectrometer constant with respect to the groove direction of the grating.

XRD patterns were recorded using a Bruker AXS D8 system with a Cu‐Kα source. AFM images were recorded on a Bruker Dimension AFM apparatus operating in tapping mode. For TEM observation, WTe_2_ was transferred onto TEM grids by using the method adopted for EDS analysis. HR‐TEM images and SAED patterns were recorded at several points of the nanobelts by using a JEM‐2100F system equipped with a probe‐aberration corrector at an acceleration voltage of 200 kV; the corresponding results were consistent with each other.

*Electrical Device Fabrication and Measurements*: For electric characterization of WTe_2_, two‐probe Ti/Au (10/80 nm) electrodes were contacted using e‐beam lithography (NBL‐NB3) and an e‐beam evaporator (Temescal FC‐2000). During device preparation, PMMA encapsulation was carried out whenever the sample was exposed to air, in order to minimize degradation; however, minimum exposure within *t* < 15 min was unavoidable. After the fabrication, the electrodes under UHV conditions at 300 °C for 1 h to improve the contact were annealed. For some devices, a 3 nm thick AlO*_x_* layer was deposited by ALD to protect the channel from oxidation.

Electrical characterization was performed in a cryogenic probe station (Lakeshore CRX‐4K) equipped with a Keithley 4200‐SCS detector, at *T* ≈ 6.5–300 K and either high vacuum to 10^−6^ Torr or atmospheric pressure, depending on the experimental conditions. For the resistance sampling mode, the electrical current density (typically, *J*_ds_ ≈ 4 MA cm^−2^) was injected every 10 s. For the electrical field sweep up to failure of the devices, the voltage sweep rate was 10 mV s^−1^. All the electrical measurements in this study were conducted using intermetallic atomic exchange along the *a*‐axis, to avoid any variations resulting from random crystalline orientation.

## Conflict of Interest

The authors declare no conflict of interest.

## Supporting information

Supplementary
