# Supplementary material for: Electrically Robust Single‐Crystalline WTe2 Nanobelts for Nanoscale Electrical Interconnects
Source: Adv Sci (Weinh). 2018 Dec 12;6(3):1801370. doi: 10.1002/advs.201801370 (PMC6364501; doi:10.1002/advs.201801370)
Supplement: Supplementary file 1 — Supplementary [file ADVS-6-1801370-s001.pdf]

Copyright WILEY-VCH Verlag GmbH & Co. KGaA, 69469 Weinheim, Germany, 2018.

ADVANCED SCIENCE Open Access

Supporting Information

for *Adv. Sci.,* DOI: 10.1002/advs.201801370

Electrically Robust Single-Crystalline WTe_2 Nanobelts for Nanoscale Electrical Interconnects

*Seunguk Song, Se-Yang Kim, Jinsung Kwak, Yongsu Jo, Jung Hwa Kim, Jong Hwa Lee, Jae-Ung Lee, Jong Uk Kim, Hyung Duk Yun, Yeoseon Sim, Jaewon Wang, Do Hee Lee, Shi-Hyun Seok, Tae-il Kim, Hyeonsik Cheong, Zonghoon Lee, and Soon-Yong Kwon**

SUPPORTING INFORMATION for

Electrically Robust Single-Crystalline WTe_2 Nanobelts for Nanoscale Electrical Interconnects

Seunguk Song[1], Se-Yang Kim[1], Jinsung Kwak[1], Yongsu Jo[1], Jung Hwa Kim[1], Jong Hwa Lee[1], Jae-Ung Lee[2], Jong Uk Kim[3], Hyung Duk Yun[1], Yeoseon Sim[1], Jaewon Wang[1], Do Hee Lee[1], Shi-Hyun Seok[1], Tae-il Kim[3], Hyeonsik Cheong[2], Zonghoon Lee[1], and Soon-Yong Kwon[1*]

[1]*School of Materials Science and Engineering and Low Dimensional Carbon Materials Center, Ulsan National Institute of Science and Technology (UNIST), Ulsan 44919, Republic of Korea*
[2]*Department of Physics, Sogang University, Seoul 04107, Republic of Korea*
[3]*School of Chemical Engineering, Sungkyunkwan University (SKKU), Suwon 16419, Republic of Korea*

[*]Correspondence should be addressed. Email to: sykwon@unist.ac.kr

- Supplementary Figures S1-S20
- Supplementary Notes 1-5 (Supplementary Table S1)
- Supplementary References 41-60

Supplementary Figures

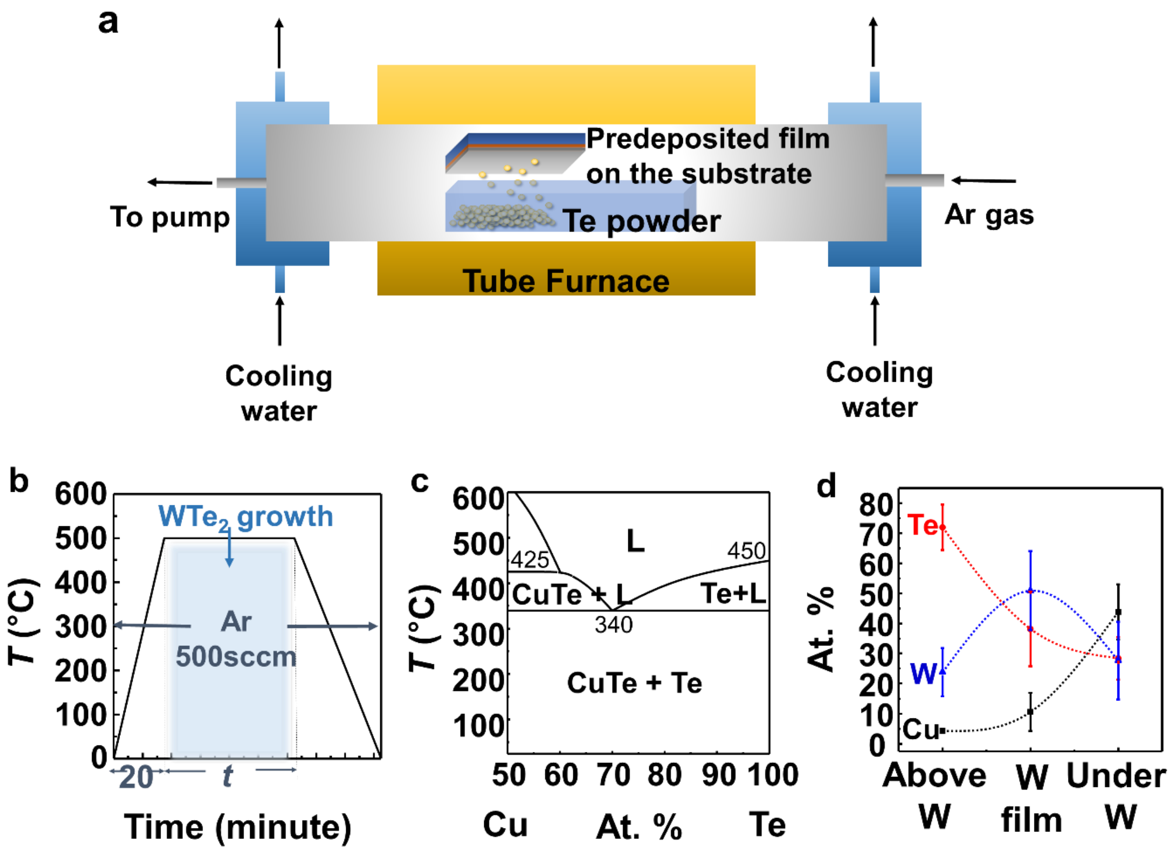

Figure S1. Growth of WTe_2 nanobelts using eutectic alloy of Cu_xTe_y. (a-c) Experimental scheme used to prepare WTe_2 nanobelts on a substrate. Generally, synthesis is conducted at T = 500 °C by tellurization of pre-deposited W-Cu layers as schemed in (a). (b) Temperatures *vs.* time during the WTe_2 growth. Growth time t, denotes the period during which the furnace is at the synthesis temperature of 500 °C. (c) Phase diagram of binary Cu-Te system.[41] (d) Atomic compositions of W layer and Cu_xTe_y above/below W layer in inset of Figure 1b characterized by EDS.

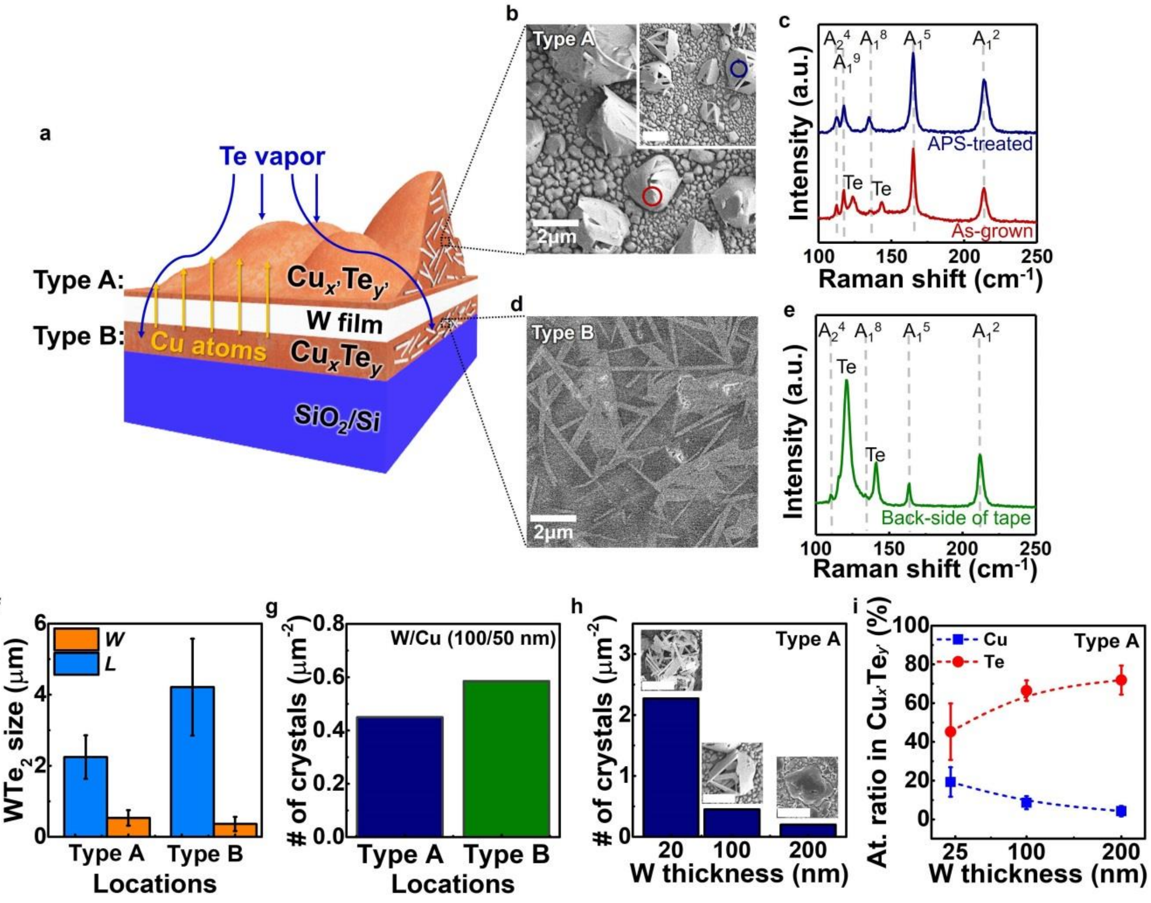

Figure S2. Behavior of liquid-like eutectic system of Cu_xTe_y to synthesize WTe_2. (a) Schematic showing the growth mechanism of WTe_2 from Cu_xTe_y(*l*) by tellurization of W-Cu-SiO_2/Si sample. (b-e) SEM and Raman characterizations of WTe_2 synthesized using pre-deposited metals of W-Cu (100/50 nm) for t = 10 min, located (b, c) above and (d, e) below W film: Type A and Type B, respectively. (b) Surface SEM image of an as-grown Type A $Cu_{x'}Te_{y'}$ sample. The inset in (b) is an SEM image of a corresponding APS-treated sample, clearly demonstrating WTe_2 growth inside $Cu_{x'}Te_{y'}$, showing an anisotropic belt-like morphology (scale bar: 2 μm). (c) Representative Raman spectra of APS-treated WTe_2 (navy) and crystals embedded in $Cu_{x'}Te_{y'}$ (wine). (d) SEM image of Type B Cu_xTe_y attached to the backside of tape after 'tape-treatment', as shown in Figure 1c. (e) Representative Raman spectrum of WTe_2 embedded in Cu_xTe_y, corresponding to the sample in (d). (f) Comparisons of WTe_2 crystal length (L) and width (W) depending on locations. The smaller L of type A WTe_2 may be attributed to limited growth due to the size of the $Cu_{x'}Te_{y'}$ droplets.[20] (g, h) Changes in number of WTe_2 crystals on samples (μm^{-2}), depending on (g) type and (h) thickness of W layer before synthesis. Inset of (h) shows SEM images of APS-treated samples grown using pre-deposited W layers of different thicknesses (scale bar: 2 μm). (i) SEM-EDS characterization of atomic ratios for Cu and Te in Type A $Cu_{x'}Te_{y'}$ for samples in (h), depending on thickness of pre-deposited W layer.

: To investigate the formation of Cu_xTe_y and WTe_2 depending on the locations above/below the W layer (Type A/B, respectively), top-view SEM and Raman spectroscopy analyses were performed. In Figures S2c and S2e, all the Raman spectra exhibited strong WTe_2 vibrational signals. The both Cu_xTe_y-containing as-grown and tape-backside samples showed two vibrational modes at ~123.0 and ~142.5 cm^{-1}, accompanying the signals corresponding to WTe_2, which may be attributed to the precipitation of Te in Cu_xTe_y during cooling. The size and morphology of Type A WTe_2 depended on the shape of the $Cu_{x'}Te_{y'}$ matrix. As shown in Figures S2b and S2f, the average nanobelt length for Type A was shorter than that for Type B, due to the limited size of the $Cu_{x'}Te_{y'}$ matrix. Further, the crystals along the $Cu_{x'}Te_{y'}$ droplet were bent compared to the flat Type B or tape-treated WTe_2 crystals.

These Type A $Cu_{x'}Te_{y'}$ droplets above W were formed by the diffusion of Cu through the W layer (probably through defects such as grain boundaries) as shown in Figure S2a. The diffused Cu could contact the Te vapour on top of the W layers, forming $Cu_{x'}Te_{y'}$, but the amount of Cu inside the droplets was lesser than that below W, *i.e.*, where it originated from (refer to the EDS analysis in the inset of Figure 1b). This may induce WTe_2 nucleation density difference due to changes in the atomic mobility in the liquefied matrix depending on the amount of Cu. Specifically, a smaller amount of Cu in the binary Cu-Te system results in a higher melting point, as indicated by the phase diagram (Figure S1c), and a lower atomic diffusivity at a certain growth temperature T. Accordingly, there were fewer Type A WTe_2 crystals than Type B ones, as summarized in Figure S2g.

To verify this mechanism, we modulated the atomic amount of Cu in the matrix. As shown in Figure S2i, the Cu amount in $Cu_{x'}Te_{y'}$ decreased with increasing thickness of the pre-deposited W layer of W-Cu-SiO_2/Si, attributed to the changed diffusion length. This successful modulation of the Cu amounts allowed remarkable changes in the WTe_2 nanobelt nucleation density via atomic mobility variations. Figure S2h shows how the nucleation density varied as the pre-deposited W layer thickness differed, analysed using SEM images (inset of Figure S2h). Dense WTe_2 nanobelts were clearly shown in the sample grown using the thin W layers (20 nm); however, as the thickness of the W layers increased (100 or 200 nm), it became difficult to observe distinct nanobelts.

This mechanism was further validated by the fact that the Ni-Te binary system (instead of Cu_xTe_y) with higher eutectic temperature hindered the formation of WTe_2 crystals.[20]

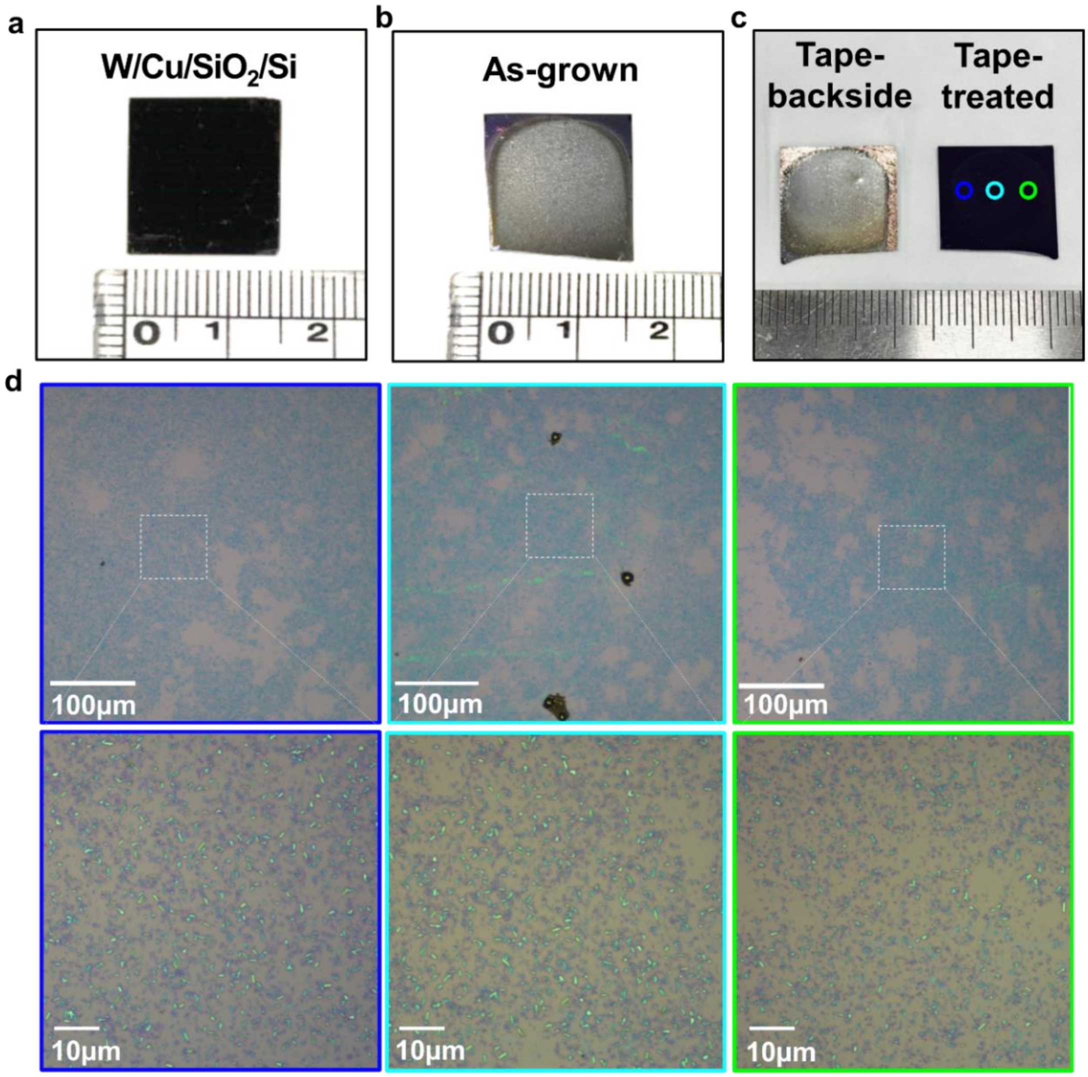

Figure S3. Tape-treated WTe_2 prepared directly on SiO_2/Si. Photographs of samples (a) before growth, (b) just after growth, and (c) prepared by tape-treatment. (d) The OM images of the tape-treated sample, showing the uniform distributions of WTe_2 crystals on SiO_2/Si substrate. The color differences of WTe_2 nanobelts in the images are attributed the thickness differences. Approximately, WTe_2 thicker than ~20 nm looks bright green. The captured positions in the tape-treated samples are marked in (c) using different colors.

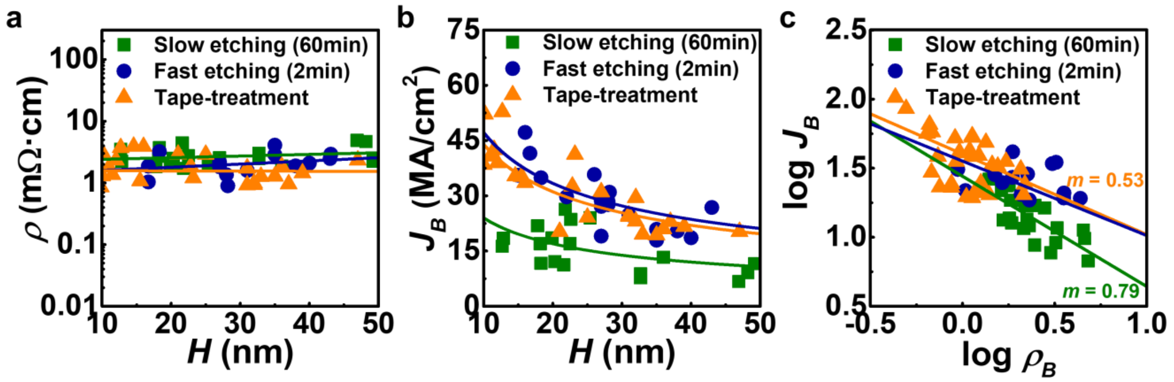

Figure S4. Electrical characterization of WTe_2 nanobelts prepared by different by-product removal methods: chemical etching and tape-treatment. (a) Low-bias (V_{ds} = 0.3 V) resistivity (ρ) and (b) high field current density (J_B) at breakdown *vs.* thickness (H) of WTe_2 prepared by varied procedures to remove as-reacted by-products like W and Cu compounds. The linear curves and the self-heating model (see Supplementary Note 3 in detail) are fitted and shown in (a) and (b), respectively. The samples treated by a 1 M APS etchant for 1 h (slow etching, green square) had the highest ρ and lowest J_B on average. On the other hand, the samples that were etched for 2 min by 30 M APS solution (fast etching, navy circle) and were tape-treated (orange triangle) had lower resistant properties as well as superior current-carrying capacity. (c) Comparisons of logarithm J_B–ρ_B plots of WTe_2 depending on varied removal conditions for W- and Cu-compounds. The extracted m, using a power law ($J_B = k\rho_B^{-m}$) for each condition, is noted. The slow-etched WTe_2 had the highest m, indicating fast, defect-induced breakdown. This degraded performance of etched samples may be attributed to the oxidation process of WTe_2 while being exposed to an APS water-containing solution for a long time. Consequently, the tape-treatment or fast etching process is suitable for WTe_2 crystals to sustain their intrinsic electrical properties.

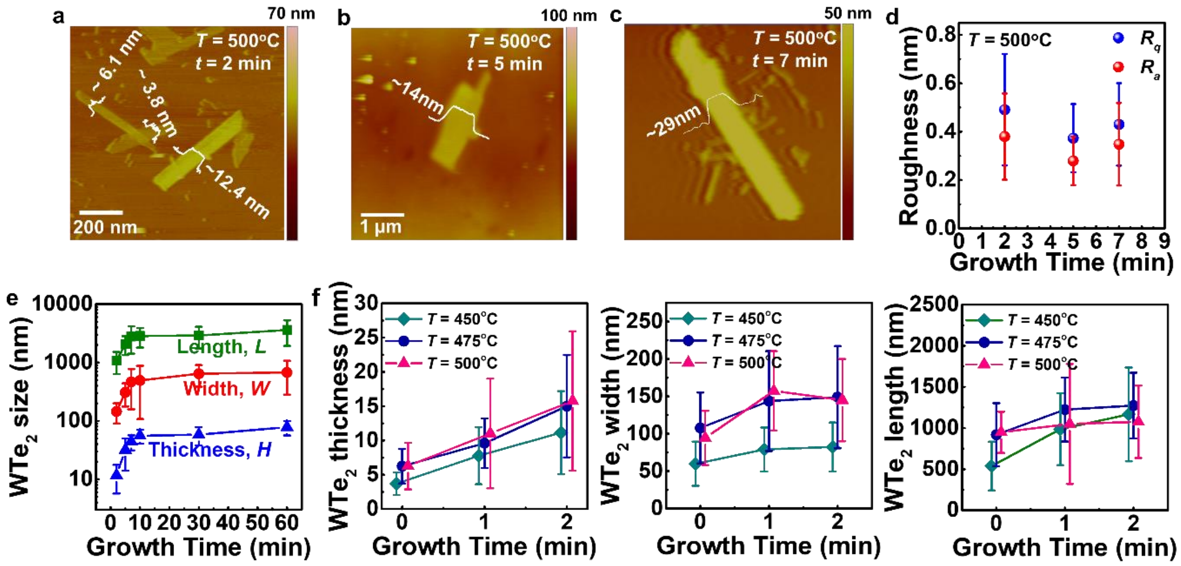

Figure S5. Growth parameters-dependent dimensions' evolution of tape-treated WTe_2 crystals using AFM analysis. (a-c) Representative AFM images of WTe_2 nanobelts grown for t = (a) 2, (b) 5, and (c) 7 min, respectively. (d) Averaged root mean squared (R_q) and arithmetic average roughness (R_a) of WTe_2 crystals extracted from captured AFM images, which is below the interlayer distance of WTe_2. This smooth property of van der Waals (vdW) metals may lessen the possibility of diffusive carrier scattering. (e) Growth time (t)-dependent dimensions' evolution of tape-treated WTe_2 grown at T = 500 °C. (f) Growth temperature (T)- and time (t)-dependent evolution of dimensions of WTe_2 sorted according to thickness (H), width (W), and length (L) (from left to right).

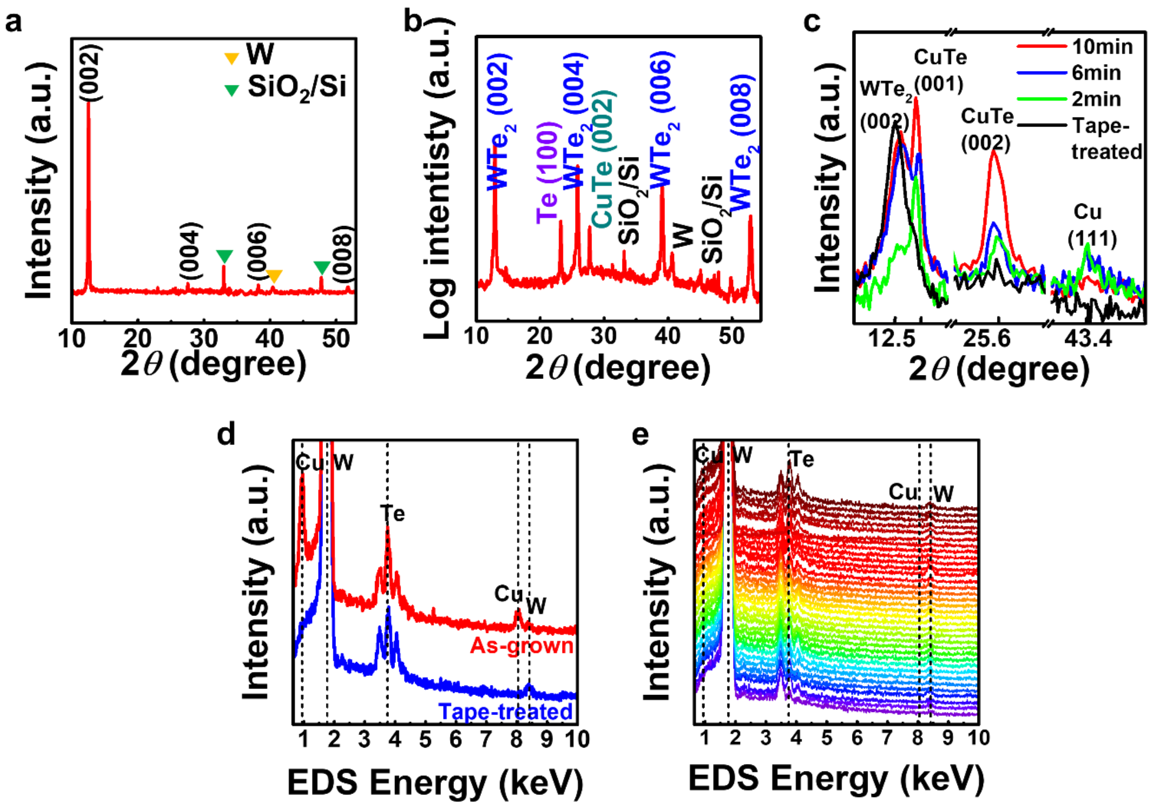

Figure S6. Complete removal of Cu from WTe_2 using the tape-treatment, analyzed by XRD and SEM-EDS. (a) XRD pattern of a tape-treated sample, having WTe_2 (002*l*) peaks without any Cu-related traces. (b) Typical XRD patterns of as-reacted sample grown for t = 60 min, showing CuTe(00*l*) peaks as well as Te(100). (c) XRD patterns of as-grown samples, magnified to identify WTe_2(002), CuTe(00*l*), and Cu(111) peaks, depending on growth time. After peeling off byproducts (tape-treatment), Cu-related peaks disappear. (d) EDS spectra of as-grown and tape-treated sample. The Te peaks at ~3.77 and W peaks at ~8.40 keV are clearly identified in both as-grown (red) and tape-treated (blue) samples' spectra. Meanwhile, Cu peaks at ~0.93 and ~8.04 keV are absent in the tape-treated sample's spectrum (blue). (e) EDS spectra of 30 different WTe_2 crystals on a tape-treated sample, without Cu peaks at 0.93 and 8.04 keV.

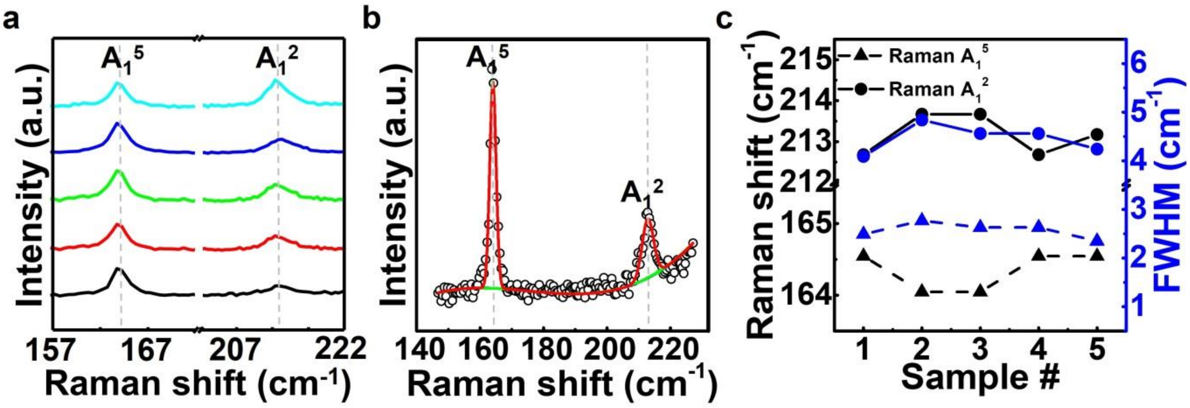

Figure S7. Zoomed-in Raman full-width-at-half-maximum (FWHM) and peak positions for WTe_2 A_1^2 and A_1^5 modes. (a) Resized five Raman spectra of WTe_2 nanobelts corresponding to Figure 1c. (b) Representative Raman spectrum of A_1^5 and A_1^2 vibrational signals (black circles) fitted with Lorentzian peaks (red). The solid green curve is the base line for fitting. (c) Raman peak positions and FWHMs of A_1^5 and A_1^2 modes from five different nanobelts were extracted by fitting. Since small strain or doping can induce observable change in Raman signals, we systematically characterized the spectra by fitting to find the exact peak positions and FWHM. Here, we could not find considerable change for the measured crystals as well as any difference from references for the exfoliated samples.[22]

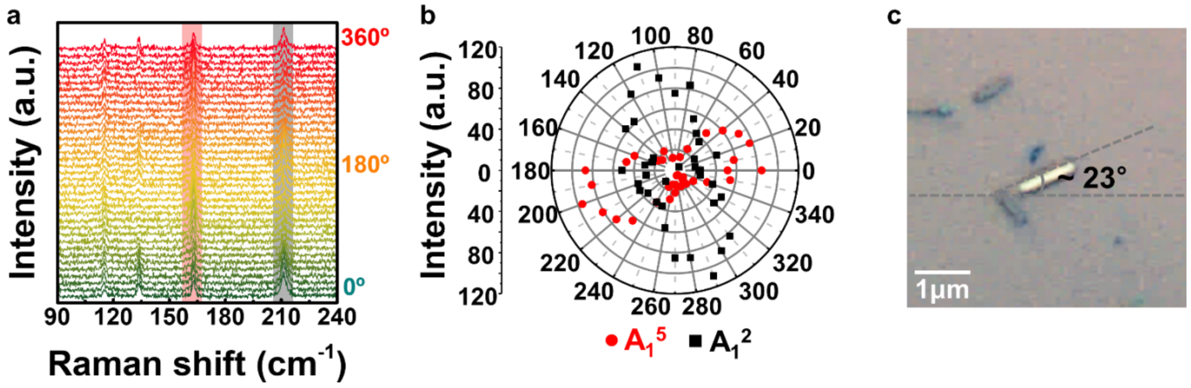

Figure S8. Polarized Raman characterization of tape-treated WTe_2 nanobelts using 632.8 nm laser as a light source. (a) Polarized Raman spectra of a tape-treated WTe_2 nanobelt shown in (b) as a function of polarized angle. The polarization angles are determined with respect to a horizontal line in (c). (b) Plot showing fitted Raman intensities of A_1^2 and A_1^5 depending on polarized angle in (a). The maximum intensities occur near the polarized angles of ~23 ± 90*n*°, which corresponds to the tilted angle of a WTe_2 nanobelt in (c), the OM image.

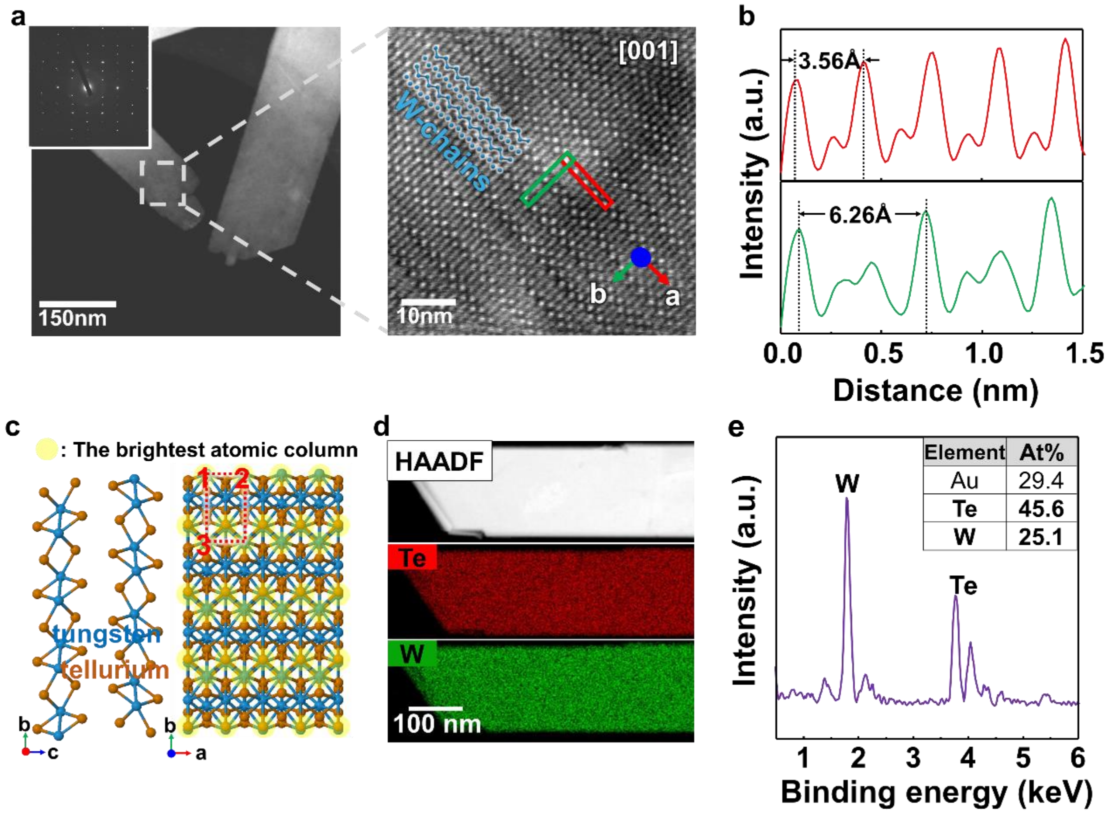

Figure S9. TEM analysis of WTe_2 crystals prepared by tape-treatment. (a) (left) Low-magnification TEM image of WTe_2 nanobelts. Inset of left image shows corresponding SAED pattern of the single crystal. (right) Atomic-resolution STEM image of a WTe_2 nanobelt at the marked point. The bright spot columns (*a*-axis) indicate W atomic arrangement forming a zig-zag chain along the longest direction of the nanobelts. (b) Line intensity profiles of the regions marked by red and blue rectangles in (a). The distances between atoms (d_{12} = 3.56 Å and d_{13} = 6.26 Å) correspond to the simulated results in (c). (c) An image showing atomic structure of bilayer WTe_2 obtained by computational simulation. The predicted brightest atomic column in STEM image of Figure S9(a), right, is indicated by a yellow circle. Since the STEM image contrast is proportional to atomic number ($\sim Z^{1.7}$), heavier atom and thicker region of W chains appears bright in the STEM image. The WTe_2 unit cell is also displayed as a dotted box, and the calculated distances between the marked points are d_{12} = 3.50 Å and d_{13} = 6.28 Å. (d, e) TEM-EDS analysis of a WTe_2 crystal. (d) EDS mapping of a WTe_2 crystal, showing the uniform distribution of each atom across the nanobelt. (e) EDS spectrum of a WTe_2 crystal. Au is detected from the TEM grid.

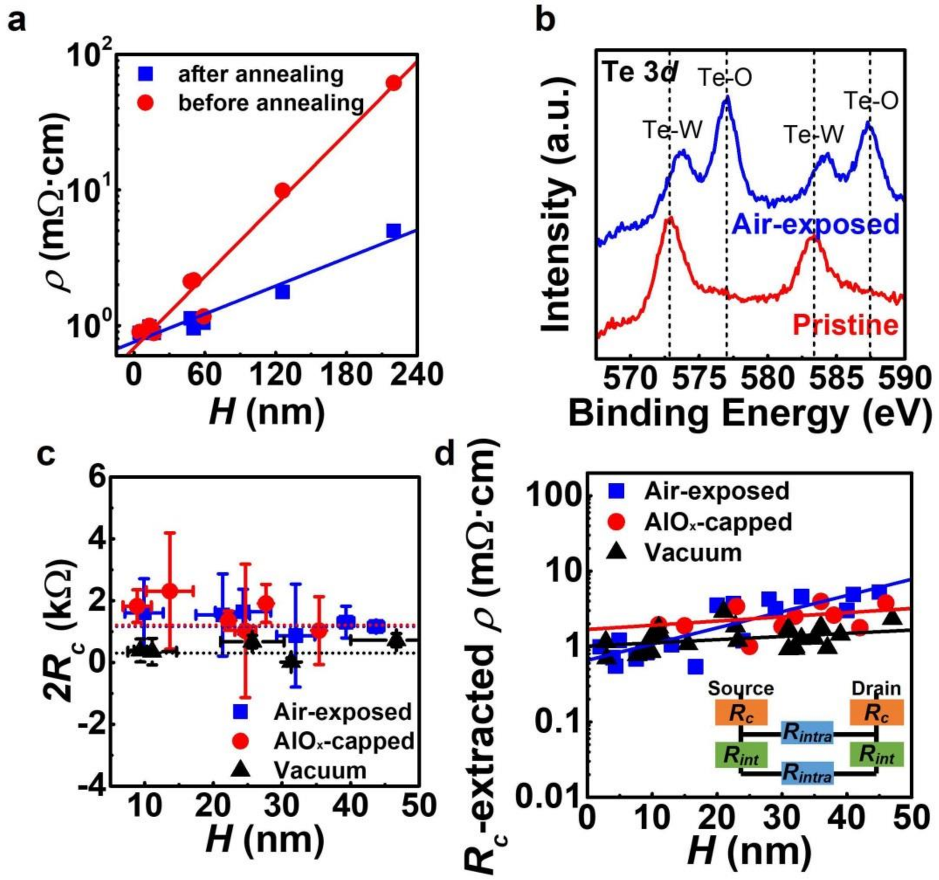

Figure S10. Low-field electrical properties of tape-treated WTe_2 nanobelts. (a) Annealing effect on the WTe_2 devices with varied thickness at low electric field ($F \approx 4$ kV/cm), showing the decrease in resistivity (ρ) especially for the thicker channel after heating in UHV ambient for 1 h at 300 °C. (b) XPS spectra of air-exposed and pristine WTe_2 showing the Te *3d* core level region, where Te-O bond appears in a sample under air ambient. (c) Normalized contact resistance (R_c) *vs.* thickness (H) characteristics extracted from two-terminal resistance depending on channel dimensions (L/W). The contact resistances also showed that the resistant nature could be affected by ambient environment. The two-terminal contact resistances[42] were extracted as follows: $R = \rho_{int}\frac{L}{W} + 2R_c$ where ρ_{int} is the interlayer resistivity (Figure S12). (d) R_c-extracted ρ as a function of H. The positive slope in (d) indicates interlayer-resistant properties of the channel, which is also shown under the R_c-included condition (Figure 2b). The positive slope which is irrelevant to R_c might be attributed to lower contact resistant natures, *i.e.*, 6.6 ± 0.5% R_c compared to entire R for the vacuum-samples on average.

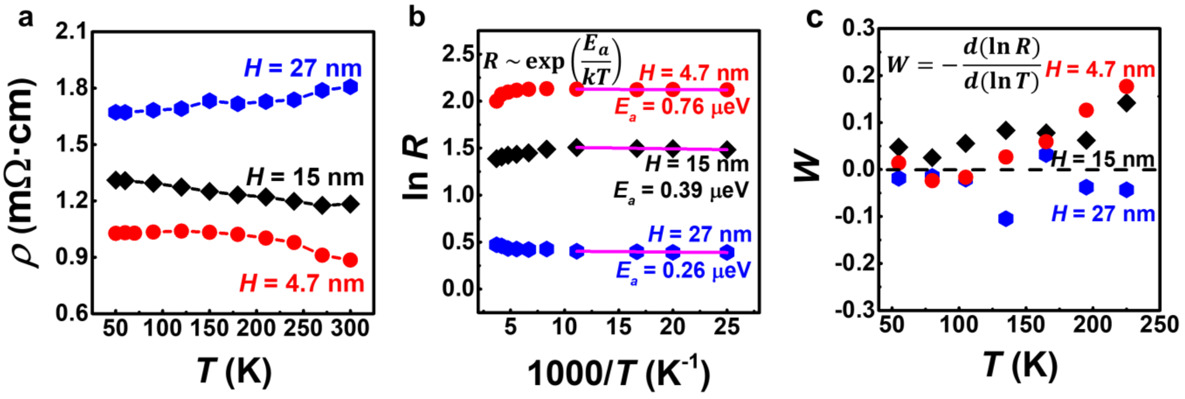

Figure S11. Temperature-dependent electrical characteristics of WTe_2 nanobelts. (a) Temperature-dependent resistivity (ρ) of WTe_2 devices with different channel thickness. Resistance increases as temperature decreases (*i.e.*, a positive temperature coefficient of resistivity;[43] TCR $\propto$ ($d\rho/dT$) > 0) for the thinner WTe_2 (H <15 nm), showing typical Anderson localization effect.[44] (b) Arrhenius plot ($ln\ R$ – $1000/T$) using the data from (a). The degree of disorder potential (E_a) can be evaluated by fitting the resistance (R) into an Arrhenius equation as $R \sim \exp(E_a/k_BT)$, where k_B is the Boltzmann constant, and E_a is the thermal activation energy, which is the potential between the Fermi level E_f at localized states and the mobility edge E_c.[31,45] The obtained positive activation energy (E_a) for thinner channel (H <15 nm) shows the insulating behavior at high temperature regime, although the value is smaller than the thermal energy $k_BT \approx 25$ meV at T = 293 K. On the contrary, the negative activation energy (E_a) is shown at low temperature regime, indicating the metallic characteristic. (c) Reduced activation energy ($W = -d(ln\ R)/d(ln\ T)$) as a function of temperature with a positive slope along all the T, which is a signal of metallic charge transport.[31,46] This indicates that the intrinsic metallic behavior lasted, even at 4.7-nm-thick WTe_2 with a negligible disorder effect, although the devices were exposed to air unintentionally during the fabrication process.

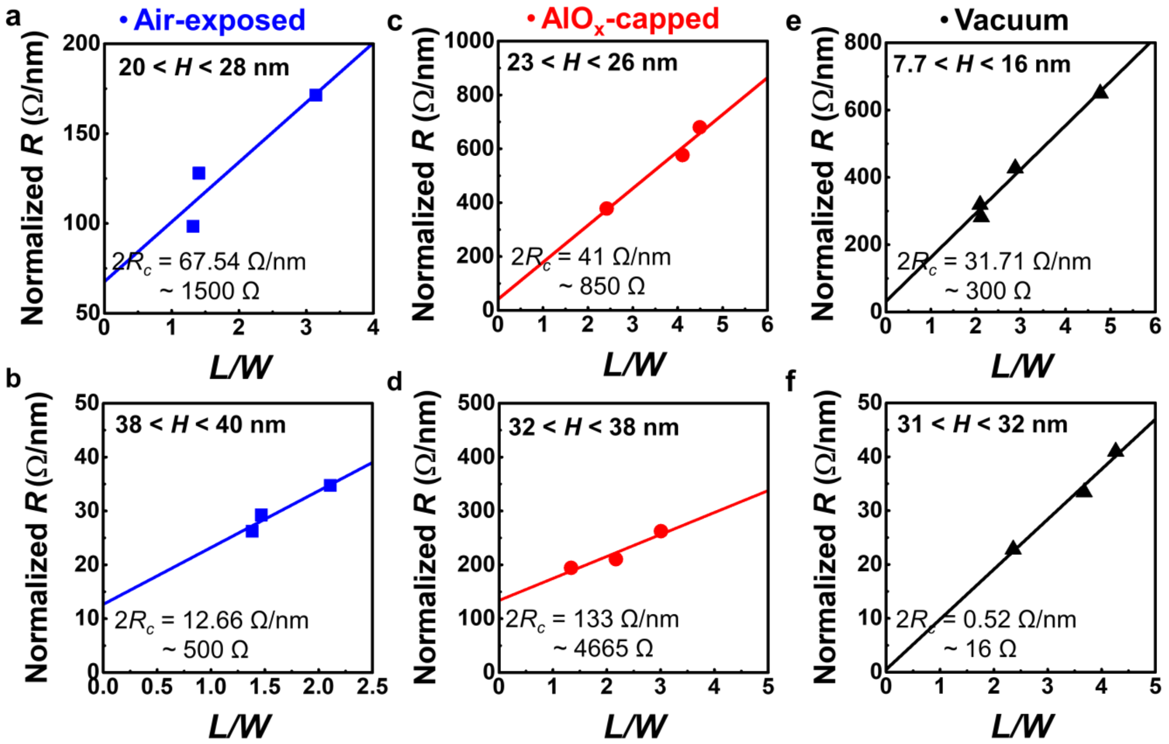

Figure S12. Extraction of contact resistance (R_c) **of WTe_2 interconnect.** Using the relation[42] $R = \rho_{int}\frac{L}{W} + 2R_c$, where ρ_{int} is the interlayer resistivity, L is channel length, and W is channel width, the contact resistance R_C could be extracted. The plots of L/W *vs.* normalized resistance (R) by channel thickness (H) is used to calculated R_C for the samples (a, b) under air ambient, (c, d) with a capping layer of AlO_x, and (e,f) under vacuum. The estimated R_C is noted in each plot.

: To calculate R_c, we used data from the WTe_2 samples with similar H because the resistivity showed a H-dependent behavior owing to the existence of interlayer resistance (Figure 2b). The R_c values are normalized to H to increase the accuracy of this calculation. Note that, to show the validation of this R_c calculation method, we tried to compare these two-terminal-R_c to the R_c extracted by the transfer-length method (TLM), as shown in Figure S13. Roughly, the vacuum-samples had $R_c \approx 300$ Ω for the samples with H in the range of 8-16 nm, whereas the air-exposed samples had 2-5 times higher R_c. The R_c values of vacuum-samples were 3-6 times lower than those of the single-crystalline, exfoliated WTe_2 flakes ($R_C \approx$ 1-2 kΩ),[14] attributable to the edge contact using *1D* geometry of our tested nanobelts rather than to the top contact that had potential barrier for the carriers to overcome formed by the vdW gap at the interface between the channel and electrodes.

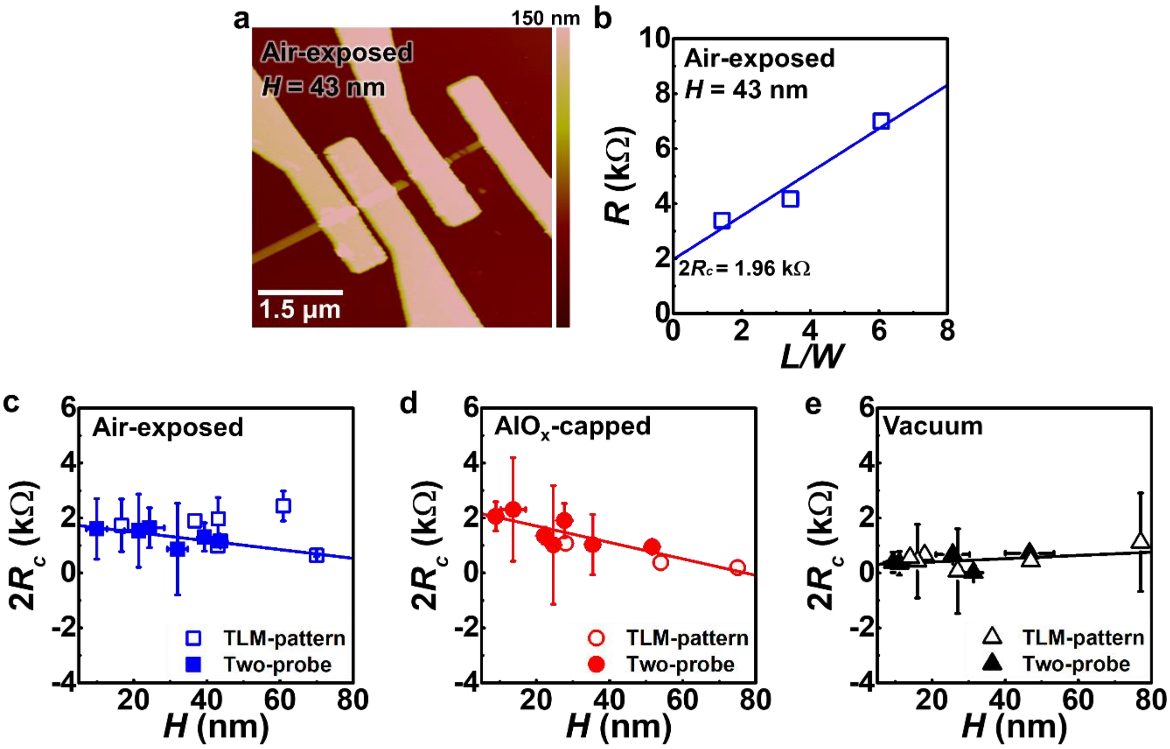

Figure S13. Calculation of contact resistance (R_c) **of** WTe_2 **using TLM-devices.** (a) A representative AFM image of a TLM-device, having a 43 nm-thick WTe_2 channel. The image was taken after the voltage sweep until the electrical failure under the air-exposed condition. (b) Corresponding electrical resistances (R) as a function of the TLM channel dimensions (L/W) were used to extract the contact resistance (R_c). The calculated $2R_c$ was 1.96 kΩ for the device in (a). (c-e) Comparison of $2R_c$ values calculated by TLM (blank) and two-terminal model (solid) under different ambient conditions: (c) under air-exposure, (d) with AlO_x-capping layer, and (e) under vacuum. The linear curve is fitted for two-terminal-R_c to provide a guide for eyes. The trend of R_c extracted by both two-probe measurements and TLM seems to correspond with each other.

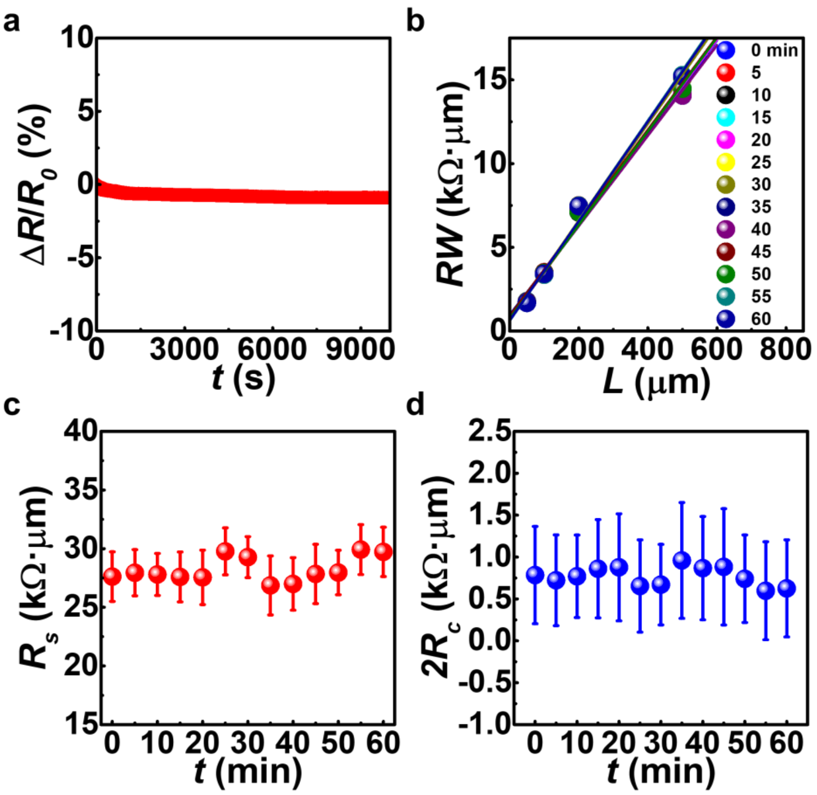

Figure S14. Uniform electrical resistance of Ti/Au contact pad regardless of time (*t*) under air exposure. (a) Resistance evolution ($\Delta R/R_0$) of a two-terminal Ti/Au channel device sampled every 10 s under V_{ds} = 0.3 V. The Ti/Au channel was also contacted by Ti/Au. (b-d) TLM characterizations of a Ti/Au-contacted Ti/Au channel device. (b) Resistance of the TLM devices *vs*. channel length (L), recorded every 5 min. The extracted sheet resistance (R_s) and contact resistance (R_c) from this device as a function of exposure time are shown in (c) and (d), respectively. There were not many significant changes in the resistances of the air-exposed Ti/Au devices, indicating that the resistance variations in the WTe_2 interconnect devices under different ambients described in the main text (Figure 2c) are not contact-limited properties.

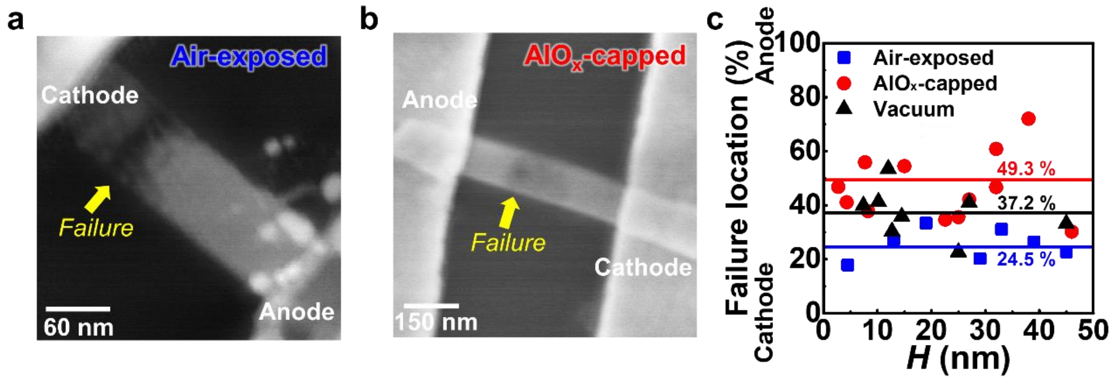

Figure S15. Electrical breakdown (*i.e.*, void formation) in WTe_2 channels by voltage sweep. (a, b) Representative SEM images of WTe_2 devices captured just after the electrical failure. The failure points of (a) AlO_x capped- and (b) air exposed-devices indicate that the electrical breakdown occurred within the channel, not at the electrode. (c) Plot of failure point location *vs.* channel thickness (H) in WTe_2 nanobelts under different ambient conditions: under air-exposure, under vacuum, and with AlO_x-capping layer.

: In our WTe_2 devices, the change in the failure point location strongly depends on the isolation of air and prevention of atomic migration on the surface, which vary strongly with ambient conditions and to a lesser extent with sample thickness (H) and contact resistance (R_c). As the currents flows from the cathode to anode, the electron-wind force ($F_{wd} = Z^*_{wd}eF$ where Z^*_{wd} is an effective charge number showing the effect of momentum transfer between diffusing atoms and electrons[1,4]) is first applied near the cathode, enabling the displacement of atoms (electromigration). Contrary to this, the Joule heating induced failure is caused by the elevated temperature of the WTe_2 devices to T_m, which WTe_2 cannot withstand. Hence, a failure could occur at a location regardless of the location of the application of F_{wd} with respect to the cathode.[47] In other words, tolerating the atomic displacement driven by electromigration is important for a breakdown away from the cathode.

Under air exposure, the effect of F_{wd} on the breakdown is even greater because the WTe_2 lattice becomes unstable in the presence of oxygen. According to previous studies on the transition metal chalcogenides (TMCs),[19,40,48] oxygen-related species can easily be absorbed on the surface causing instability to the atomic structure. This creates chalcogen-oxygen (*i.e.*, Te-O) pairs that can be desorbed from the surface, leaving a vacancy at a chalcogen site. In the case of WTe_2, the oxygen adsorption process is even easier than in any other TMC (*i.e.*, the highly exothermic process); [19,48] the Te atoms float to the top of the surface leading to structural degradation.[19] Therefore, the oxygen-related vacancy formation and diffusion at the surface increase the rate of the electromigration. As we have characterized in the manuscript, the AlO_x-capping layer can effectively hinder the oxidation of WTe_2, which in turn suppresses such defect-induced electromigration, leading to relatively random breakdown locations.

Nevertheless, the isolation of air is probably not sufficient to suppress the electromigration because the failure location of WTe_2 under vacuum is observed to shift a little towards the anode, unlike in the case of the AlO_x-capped ones. Hence, there is an additional role of the AlO_x capping layer to restrain the electromigration: We propose that the

AlO_x capping layer physically prevents atomic migration on the surface. Since the passivation layer is physically constrained to the WTe_2, the diffusion of vacancies or atoms along the surface can be effectively blocked, especially considering the unstable Te layers that are freely exposed to surface. A previous study also reported that the failure location was away from the cathode for an encapsulated Cu interconnects,[49] which supports our assumption. Furthermore, in the case of the vacuum-WTe_2, although there is no oxidation-related vacancy formation, there still exists free surface where vacancies are movable (*i.e.*, diffusion of vacancies) under the electrical stress, which causes failure due to a slight electrical wind force.

The role of the AlO_x capping layer on electromigration is further displayed in the caption of Figure S18.

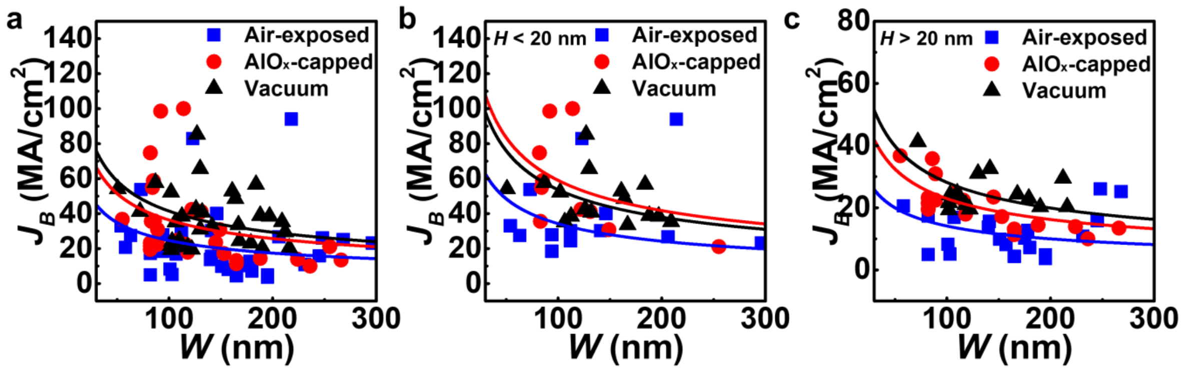

Figure S16. Electrical breakdown current density of WTe_2 interconnects depending on channel width. (a) Breakdown current density (J_B) of the measured WTe_2 nanobelts *vs*. width (W). (b-c) Corresponding J_B-W plots for (b) the thinner (H <20 nm) and (c) thicker samples (H >20 nm). Solid lines indicate curves fitted to the *1D* heat dissipation model ($J_B \propto W^{-1/2}$). The value of J_B seems to rely on H (Figure 3c) rather than W in the nanobelts shown in (a) because the rate of change in H (*i.e.*, for $2 < H < 50$ nm) is a bit larger than the variation in W (*i.e.*, for $50 < W < 300$ nm). However, as can be seen in each plot using the data from WTe_2 with similar W values (b and c), the value of J_B agrees well with the model.

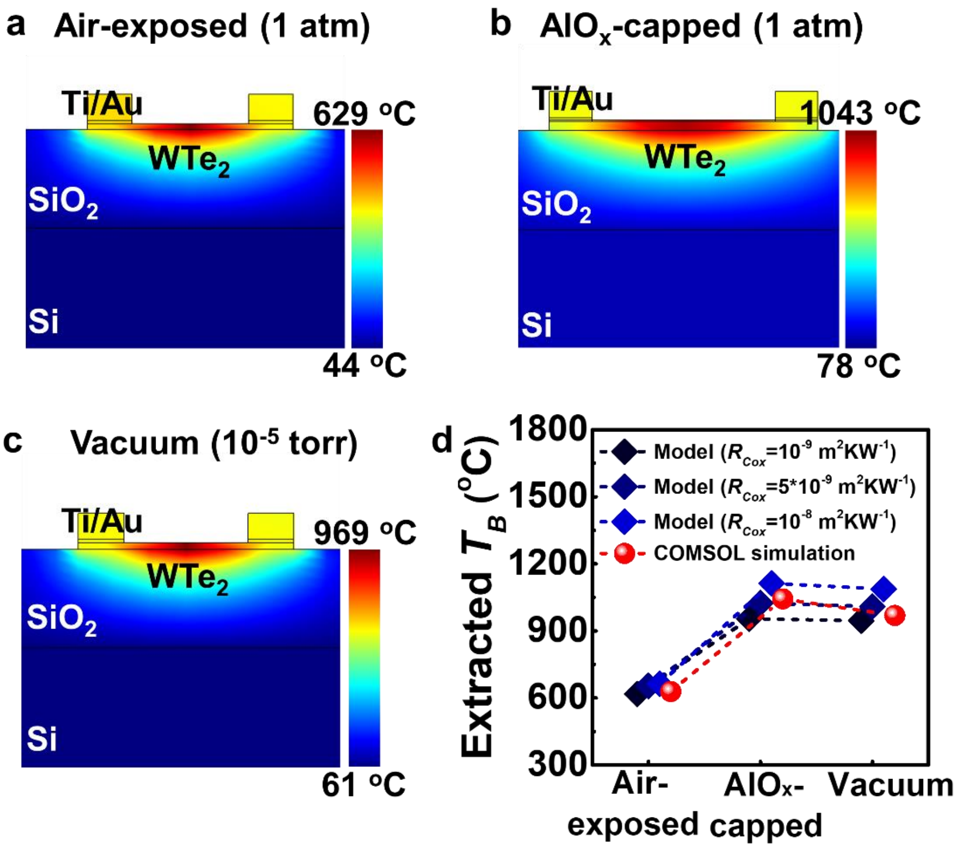

Figure S17. Finite-element simulations of self-heating at WTe_2 interconnect structure to calculate temperature during an electrical breakdown. Temperature gradient profiles at WTe_2 tested devices (a) under air exposure, (b) with AlO_x-capping layer, and (c) under vacuum in a non-convection system are shown, which were calculated by finite-element simulation performed using a COMSOL Multiphysics 4.3b modeling software. For the computations, average values for input power and geometries were selected and the surrounding air convection was set depending on each ambient condition. For example, the parameters used for calculation for the AlO_x-capped devices were P = 1.326 mW, L = 553 nm, W = 129 nm, and H = 28 nm. We assumed that air convection existed. (d) Comparisons of T_B calculated by thermal transfer model (blue diamond) and COMSOL (red circle). For the heat transfer model, we showed T_B extracted using different thermal contact resistance values between WTe_2 and SiO_2 (R_{Cox}) to confirm the validity of using R_{Cox} in the thermal models (Supplementary Note 3). The deviation of T_B calculated with different R_{Cox} (10^{-8} and 10^{-9} m^2KW^{-1}) was around 100-150 °C.

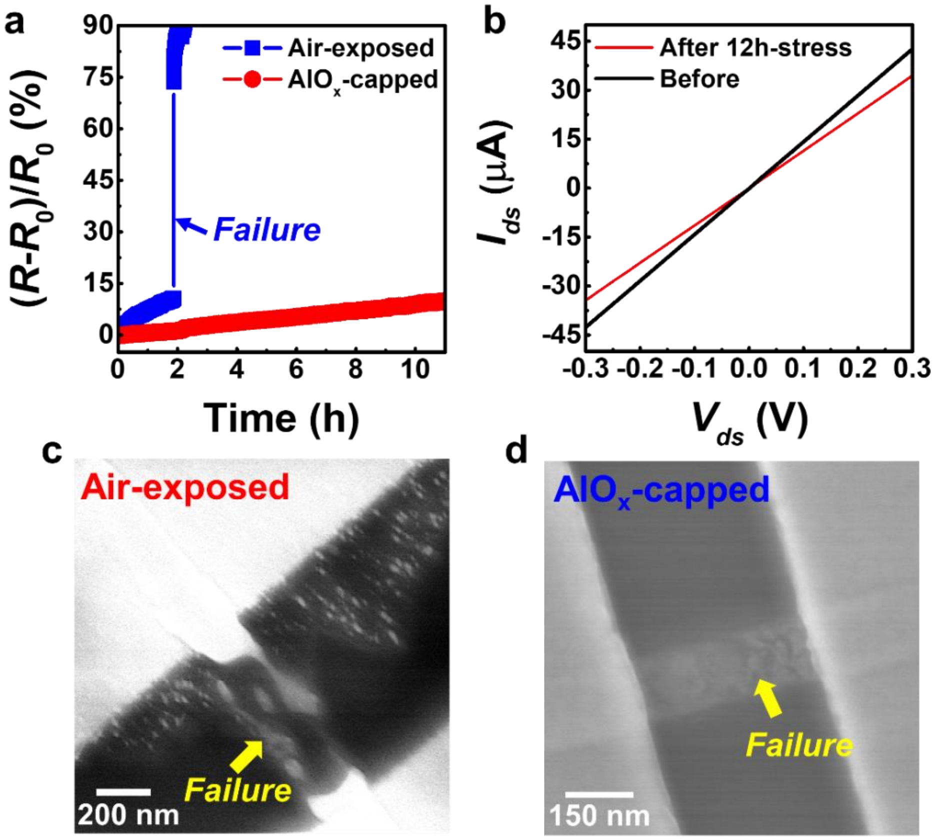

Figure S18. Mean-time-to-failure test of AlO_x-capped device to validate the role of passivation to suppress electromigration. (a) Percent change of resistance, $(R\text{-}R_0)/R_0$ of air-exposed and capped devices as function of stress time, while sampling at DC-bias of 0.3 V. Air-exposed device showed abrupt increase in resistance, indicating the failure happened. (b) Current *vs.* voltage (I_{ds}-V_{ds}) curve showing the Ohmic behavior, even after current stress for 12 h. (c) SEM image of a device under air shows severe degradation of the channel, though the capped-channel remains even after the high-bias stress for 12 h, as shown in (d).

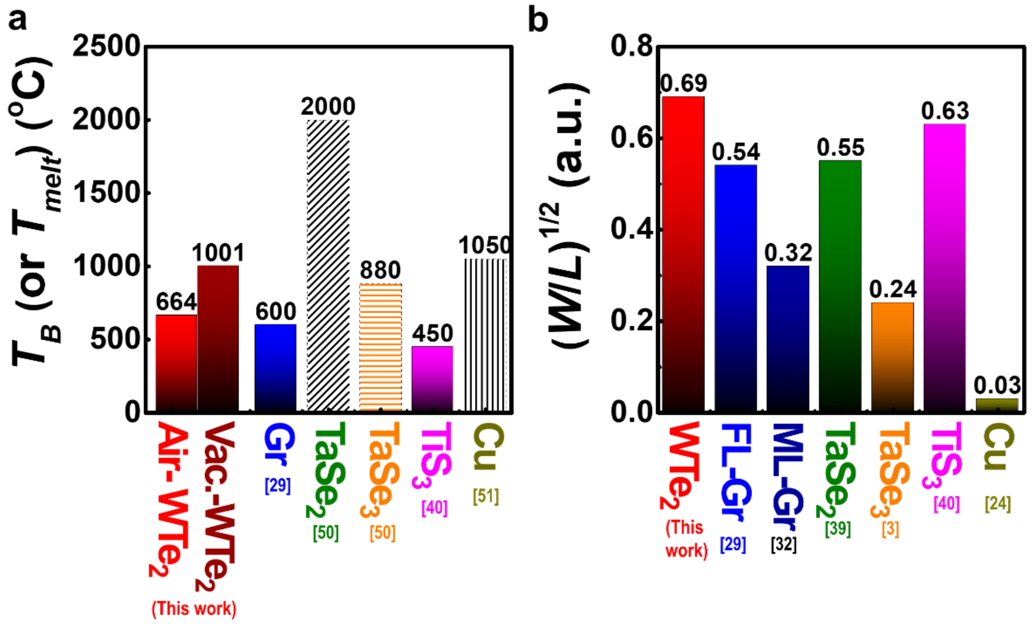

Figure S19. High durability in WTe_2 against electrical breakdown compared to other materials. (a) Comparison of extracted maximum temperature at breakdown (T_B) of WTe_2, graphene (Gr),[29] and TiS_3.[40] For tantalum selenide[50] (*i.e.*, $TaSe_2$ and $TaSe_3$) and Cu[51] the melting temperature (T_{melt}) is displayed (patterned box) owing to less information on T_B by voltage sweep. (b) Comparison of channel dimensional value of $(W/L)^{1/2}$ that linearly affects heat transport rate (g). The $(W/L)^{1/2}$ are calculated using data from each reference for few-layered *FL*-Gr,[29] *ML*-Gr,[33] $TaSe_2$,[39] $TaSe_3$,[3] TiS_3[40] and Cu.[24] Since the substrate of the compared materials (except Cu) was 300 nm-thick SiO_2 on Si, only the materials' phonon conductance is related to g.

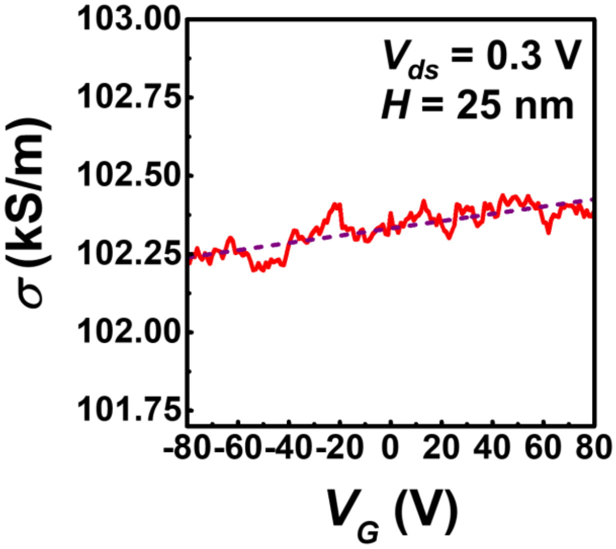

Figure S20. *n*-type behavior of WTe_2 FET at room temperature. The figure clearly shows that major carrier in tape-treated WTe_2 nanobelts is the electron, because the conductivity increases as electrostatic force is swept. The back-gate voltage was applied using heavily doped *p*-Si. Although the device had major carrier of electron, the gate dependence is significantly small because of semi-metallic WTe_2's negative band-gap compared to semiconductors.

Supplementary Note 1

To prepare WTe_2 nanobelts directly on a SiO_2/Si substrate, we manipulated the eutectic alloy (Cu_xTe_y)-assisted synthesis method. At growth temperature T = 500 ^oC, the pre-deposited sample of W-Cu-SiO_2/Si reacted with Te vapour, forming a $Cu_xTe_y(l)$ eutectic alloy near the W layer. Since the T was higher than the eutectic point (≈ 340 ^oC) of the Cu-Te binary system[41] (Figure S1c), Cu_xTe_y existed as a liquid matrix. The liquid phase of $Cu_xTe_y(l)$ allowed rapid synthesis of WTe_2 by the following proposed roles: i) Te atoms existed in a liquid phase rather than in a vapor phase, which halted the Te-deficient condition. Considering high vapour pressure and low sublimation temperature of Te, formation of the $Cu_xTe_y(l)$ facilitated the nucleation and growth of WTe_2. ii) molten state of eutectic provided an atomically mobile condition, facilitating faster atomic diffusion and reaction of the constituent elements. For example, W atoms (or WTe_2 quasi-nucleus) could diffuse from the interface of the Cu_xTe_y/W layer into Cu_xTe_y matrix, resulting in the growth of WTe_2 nanobelts inside the liquid-like Cu_xTe_y at a high rate.

In our experiments, liquid-like Cu_xTe_y formed just below the W layer (*i.e.*, above SiO_2/Si substrate), which was possible by atomic diffusion of Te through the GBs at the polycrystalline W layer. In the cross-sectional SEM image of an as-reacted sample (left inset in Figure 1b), clearly distinct structures can be seen, which correspond to $Cu_{x'}Te_{y'}$/W/Cu_xTe_y, and SiO_2/Si substrate from top to bottom, respectively, as identified from the EDS map (right inset in Figure 1b) and atomic composition profiles (Figure S1d). By considering the above-mentioned roles of Cu_xTe_y in the synthesis of WTe_2, the locations of eutectic alloy reservoir could determine the exact position where WTe_2 would form. For instance, after the removal of Cu_xTe_y and by-products of W by tape-treatment, we could find WTe_2 crystals directly on the SiO_2/Si substrate, where Cu_xTe_y had been.

The structural analysis reveals that there is no noteworthy variance in the resultant data with respect to the pre-deposited stacking sequence of metal layers and by-product removal process. However, electrical degradation is obvious for the WTe_2 nanobelts synthesized using Cu-W-SiO_2/Si sample (Ref. 20) compared to one synthesized by W-Cu-SiO_2/Si stacking (this study). This is probably attributed to the additional requirement of APS treatment and transfer procedures for WTe_2 grown using Cu-W stacked assembly in Ref. 20, which results in exposure to water molecules and supply of electrical bias in an acidic solution. These approaches are unnecessary for the present study.

Supplementary Note 2

The importance of air-passivation in the WTe_2 devices was investigated by conducting resistance sampling as a function of time at V_{ds} = 0.3 V. We observed upsurges in the resistance of an air-exposed device with time, contrary to those under vacuum and with an AlO_x capping layer (Figure 2c), and fitted electrical data into a following relation to unveil the degradation rate of current (I_{ds}):

$$I_{ds}(t) = A - Bt + Cexp\left(-\frac{t}{\gamma}\right) \quad \text{(S1)}$$

The fitted parameters from our work were A = 257.9 μA (~3.82 MA/cm^2), B = 0.23 nA/sec (~34 A/cm^2·s), C = 9.51 μA (~0.14 MA/cm^2), and γ = 1,522 s with an R-square value of 0.9982, and the linear and exponential contributions from these parameters are plotted (inset in Figure 2c). Because the prepared WTe_2 has major carriers of electrons (Figure S20), it is acceptable to consider that the exponential decrease in current is owing to instant and reversible p-type doping by molecules' absorption like oxygen and moisture, as observed in other vdW materials.[25,26] However, as noticeable in the XPS spectrum (Figure S10b), air-induced oxidation can lead to an irreversible and linear decrease in current by shrinkage channel. This leads to the permanent degradation of current, which was quite fast for this tested WTe_2 nanobelts; the rate (B) of the WTe_2 was higher than Cu[24] (~0.62 nA/cm^2·s) and phosphorene[25] (~0.55 mA/cm^2·s), but lower than MXene[26] (~1.8 kA/cm^2·s), implying that air-passivation is critical for WTe_2 to sustain its intrinsic electrical properties.

Supplementary Note 3

The ideal *1D* heat dissipation relationships shown in the main text (Equations (1) and (2)) are: $P_B = g(T_B - T_0)L$ and $J_B = \left[\frac{g(T_B-T_0)}{\rho_B HW}\right]^{\frac{1}{2}}$, where g is the thermal conductance of the channel per unit length contacting the substrate and the electrode, T_B is the Joule heating-induced maximum temperature at breakdown, T_0 is the temperature of the ambient (~ 300 K), and ρ_B is the resistivity at failure. As indicated in the equations, a higher thermal conductance (g) causes higher input power (P_B) and higher J until breakdown, which is significantly affected by the channel area ($W\times L$) contacting to the substrate. This is because the fast heat transport through the contacted area into the SiO_2/Si substrate contributed to the overall g of the channel, as implied in Figure 3e. In detail, g is a parallel combination of spreading thermal resistance in the oxide (R_{ox}), thermal resistance from SiO_2 to Si (R_{si}), and thermal contact resistance between the channel and the oxide interface (R_{Cox}):[14]

$$\frac{1}{g} = \frac{R_{Cox}+R_{ox}+R_{si}}{W} = \frac{R_{Cox}}{W} + \left\{\frac{\pi k_{ox}}{ln\left[6\left(\frac{t_{ox}}{W}+1\right)\right]} + \frac{k_{ox}}{t_{ox}}W\right\}^{-1} + \frac{1}{2k_{si}}\left(\frac{L}{W_{eff}}\right)^{\frac{1}{2}} \qquad \text{(S2)}$$

Here, t_{ox} is the thickness of SiO_2, $W_{eff} \approx W + 2t_{ox}$ is the effective width of the heated region at the SiO_2/Si interface by the fringing effect,[29] k_{ox} is the oxide thermal conductivity expressed as $\ln(T_{ox}^{0.52})-1.687$ at a temperature $T_{ox} \approx (T_B + T_o)/2$, and $k_{si} \approx 24{,}000/T_0$ is the Si thermal conductivity, where T_0 is the ambient temperature of the device. Consequently, g is dependent on the dimensional constraints of a device channel, as we demonstrated from the relation $g \propto (W/L)^{1/2}$ in Figure 4c. This calculation for g does not contain the parameter H because the same temperature distribution along the vertical direction is assumed as the nano-thick channel is well-contacted with the substrate as a heat sink.

The range for R_{Cox} at the interface between WTe_2 and SiO_2 may be between 10^{-8} and 10^{-9} $m^2\,KW^{-1}$, similar to that other layered materials (*i.e.*, graphene[29,52] and MoS_2[53]). A previous study[14] also showed that R_{Cox} at the WTe_2-SiO_2 interface is ~10^{-8}-10^{-9} $m^2\,KW^{-1}$ depending on the crystals' thermal conductivity. Although aspects such as the interface formation process or crystal quality can affect R_{Cox}, we showed that a small R_{Cox} deviation in that range (~10^{-8} -10^{-9} $m^2\,KW^{-1}$) does not render our thermal modeling invalid, as shown in Figure S17d. For instance, the difference between T_B values calculated with varied R_{Cox} of 10^{-8} and 10^{-9} m^2KW^{-1} was around 100-150 °C, which was quite small (even similar to the standard error (~100 °C) caused by different channel dimensions) and did not affect the tendency negatively. Therefore,

in most thermal models, we used R_{Cox} of 10^{-8} m^2KW^{-1} and the model fits well into experimental data.

It should be noted that the above *1D* heat dissipation model does not include any effect from thermal contact resistance (R_T) at the interface between the channel and metal contact because the channel is 'long' enough to dissipate most of the heat vertically rather than laterally. For example, heat conduction equation with a finite R_T is suggested[14,29] as follows:

$$J_B = \left[\frac{g(T_B - T_0)}{\rho_B HW} \times \frac{\cosh\left(\frac{L}{2L_H}\right) + gL_H R_T \sinh\left(\frac{L}{2L_H}\right)}{\cosh\left(\frac{L}{2L_H}\right) + gL_H R_T \sinh\left(\frac{L}{2L_H}\right) - 1}\right]^{1/2} \quad \text{(S3)}$$

$L_H = (k_{eff}/g)^{1/2}$ is a thermal healing length along the channel and $R_T = [L_{Hm}/(k_m t_m(W+2L_{Hm}))]$, where $k_{eff} \approx k_{channel} + k_{cap}(H_{\mathrm{cap}}/H)$; here, L_{Hm} (= $(k_m t_{ox} t_m/k_{ox})^{1/2}$) is the thermal healing length of heat spreading into the metal contacts, k_m is the thermal conductivity of the metal contact, and t_m is the contact thickness. If the channel length, L, is much longer than L_H, Equation S3 reduces to $J_B = \left[\frac{g(T_B - T_0)}{\rho_B HW}\right]^m$ (recall that $sinh(x) = cosh(x) = exp(x)/2$ for $x >> 1$). Thus, for a long device ($L >> L_H$), it is acceptable to neglect the R_T effect on heat conduction, while heat sinks mainly through the underlying SiO_2 vertically. In our WTe_2 devices, the average L_H was obtained as ~100 nm (Figure 4d) by considering the parallel contribution from lateral heat flow through the channel ($k_{channel} \approx 9.03$ W/(m K) of WTe_2 along *a*-axis[54]) and existence of a capping layer ($k_{cap} \approx 4$ W/(m K) for AlO_x[14])). (The calculated L_H is similar to that of graphene, 100-200 nm.[29]) Since the channel lengths of our tested devices were at least three times longer than L_H, practical use of the reduced version of *1D* heat dissipation model is valid.

Supplementary Note 4

We calculated breakdown current density (J_B) as a function of cross-sectional area of the channels (WH) in Figure 5b using 1D heat dissipation equation and Fuchs-Sondhemer (F-S) model for surface scattering. The resistivity change by F-S model for electron-nanobelt scattering is expressed as:

$$\rho_{FS} = \rho_0 \left[2Cl_0\,(1-p)\left(\frac{1}{H}+\frac{1}{W}\right)\right] \qquad \text{(S4)}$$

, where ρ_0 is the bulk resistivity, C is the constant for rectangular geometry (~1.2), l_0 is the bulk mean free path of electron, and p is the specularity parameter that is related to the electron scattering motion (p = 1 for pure scattering without additional resistivity increment, while p = 0 for diffusive scattering). Considering the thickness-dependent resistant behavior (existence of R_{int} as mentioned in the main text), we obtained total resistivity change by using Matthiessens' rule as follows:

$$\rho = \rho_{FS} + \rho_H, \quad \text{or} \qquad \text{(S5)}$$

$$\rho = \rho_0 \left[2Cl_0\,(1-p)\left(\frac{1}{H}+\frac{1}{W}\right)\right] + \frac{d\rho}{dH}H. \qquad \text{(S6)}$$

Here, the parameters used in the calculation for F-S model were W = 250 nm, L = 500 nm, and ρ_0 = 2 mΩ·cm. The electron mean free path, l_0, was assumed to become W/4. Note that conventional interconnect material of Cu usually has l_0 of ~40 nm[3]. Finally, to obtain breakdown current density (J_B), we put ρ from Equation (S6) into Equation (2) and used the average parameters of our tested devices, in the following equation:

$$J_B = \left[\frac{g(T_B-T_0)}{\rho_0\left[2Cl_0\,(1-p)\left(\frac{1}{H}+\frac{1}{W}\right)\right]HW+\frac{d\rho}{dH}H^2W}\right]^{\frac{1}{2}} \qquad \text{(S7)}$$

Supplementary Note 5

"Post-CMOS interconnect" is required since the signaling and reliability are limited by interconnect technology as integrated circuit feature sizes are downscaled. This is due to the fact that, compared to smaller transistors, which has advantages of lower operating voltage and faster switching speed, downscaled interconnects show increased resistance and inter-metal capacitance (dielectric permittivity). We calculated how the performance of WTe_2 interconnect could be improved in percentage, compared to conventional poly-crystalline metals and other candidate vdW materials, based on signaling and reliability.

(i) Signaling

The major parameter used to evaluate signaling is RC delay, which is the delay time for an electrical signal to propagate through an interconnect and is expressed as $\tau = RC$, where R is resistance and C is the capacitance of total interconnect system.[55] Interconnect scaling would cause a notable increase in R as the polycrystalline conductor exhibits severe carrier scattering mechanisms from the surface and grain boundaries. An increased R_c value is also a problem, which is attributed to the reduced contact area. In such a case, a dielectric material with a lower k value would reduce C and increase R, thus allowing control over RC delay. However, as aggressive scaling is in progress (*i.e.*, interconnect for sub-5 nm nodes for 2021 according to ITRS,[5] the total interconnect delay would exceed that from the gate until total delay finally increases.[56]

In our manuscript, we revealed that an AlO_x-capped WTe_2 nanobelt exhibited an R_c-extracted ρ value of ~ 280 μΩ·cm (the total R value is 7.6 kΩ, including an R_c value of ~1 kΩ at the WTe_2-Au interface) for a channel with ~250 nm^2 cross-sectional area (2.5 nm thickness) and sustaining J_B = 100 MA/cm^2. Compared to this, the Cu-related interconnect can have a lower resistivity of ~7 μΩ·cm at the channel with 30 nm width, thus restraining the maximum current capacity to ~2 MA/cm^2 (Ref. 53) (Table S1). Additional sub-5-nm technology would lead to an exponential upsurge in channel resistivity for Cu, although single-crystalline WTe_2 may be affected less as described in main text. Following the scattering mechanism in Supplementary Note 4, we could calculate the increase rate in ρ for a sub-5nm interconnect compared to bulk (ρ_{5nm}/ρ_{bulk}), as shown in Table S1. Finally, this turns out that a WTe_2 nanobelt would have roughly 10-45% lower total R for sub-5nm technology, leading to 10-45% faster signaling. Certainly, any simple parallel comparison for the materials may sound

irrational, but further scaling analysis such as accurate computer simulations for a driver-interconnect-load circuit is beyond the scope of our study.

(ii) Reliability

Regarding reliability, the current density increase caused by reduced linewidth may cause interconnect breakdown, which is affected primarily by electromigration. For example, the anticipated maximum current density for interconnects in future nodes is already J_{max} >2 MA/cm^2 from 2014 onward, as suggested by ITRS.[5] However, conventional interconnect materials (*e.g.*, bulk Cu and W), which exhibit current-carrying capacity of a few MA/cm^2, cannot meet this requirement.

With this in mind, we suggest single-crystalline WTe_2 nanobelts as an alternative interconnect material, since the material could sustain the highest robustness against Joule heating-induced failure (as described in the P_B/L study) compared to other candidate materials without evidence of carrier-scatting at the linewidth with cross-sectional area down to 200 nm^2. The tested WTe_2 nanobelt was a factor 2,382 larger than P_B/L (*i.e*, 238,200% better reliability) compared to Cu (Figure 5d). Although further reliability characterizations such as Blech length (L_B) or other capping layer are desired, we believe this firmly establishes the reliability of the proposed material.

Table S1. Properties of WTe_2 as a candidate for interconnect and comparison with Cu, W, and multilayer graphene (ML-Gr).

**Material**	**$W \times H \times L$ (nm)**	**R (kΩ)**	**ρ (μΩ·cm)**	**R_c (kΩ)**	**J_{max} or J_B (MA/cm^2)**	**ρ_{5nm}/ρ_{bulk}**	**R_{5nm} for L = 500nm (kΩ)**
Cu	30 × 30 × 130 [57]	0.024 [57]	7 [57]	0.015 [57]	2 [55]	45-75 [3]	121-201
W	30 × 30 × 130 [58]	0.060 [58]	38 [56]	0.005 [56]	1 [55]		657-1,096
ML-Gr	250 × 5 × 5,000 [59]	3.8 [59]	10 [57]	1 [59]	500 [55]	~100-150 [60]*	388-578
WTe_2 nanobelt (This work)	100 × 2.5 × 600	7.6	280	0.5	100	~1	109

* Calculated by using mobility increase in graphene [60].

Supplementary References

41 A. S. Pashinkin, V. A. Fedorov, *Inorg. Mater.* **2003**, *39*, 539.

42 Z. Fei, T. Palomaki, S. Wu, W. Zhao, X. Cai, B. Sun, P. Nguyen, J. Finney, X. Xu, D. H. Cobden, *Nat. Phys.* **2017**, *13*, 677.

43 R. C. Doty, H. Yu, C. K. Shih, B. A. Korgel, *J. Phys. Chem. B* **2001**, *105*, 8291.

44 L. Wang, I. Gutierrez-Lezama, C. Barreteau, N. Ubrig, E. Gianini, A. F. Morpurgo, *Nat. Commun.* **2015**, *6*, 8892.

45 M. Pasek, G. Orso, D. Delande, *Phys. Rev. Lett.* **2017**, *118*, 170403.

46 J. Halim, S. Kota, M. R. Lukatskaya, M. Naguib, M.-Q. Zhao, E. J. Moon, J. Pitock, J. Nanda, S. J. May, Y. Gogotsi, M. W. Barsoum, *Adv. Funct. Mater.* **2016**, *26*, 3118.

47 H. V. Nguyen, C. Salm, B. Krabbenborg, K. Weide-Zaage, J. Bisschop, AJ. Mounthaan, F. G. Kuper, *Effect of thermal gradients on the electromigration life-time in power electronics* in *2004 IEEE Int'l Reliability Phys. Symp. Proceedings*, IEEE, Phoenix, USA **2004**, pp. 619-620.

48 C. Naylor, W. M. Parkin, Z. Gao, H. Kang, M. Noyan, R. B. Wexler, L. Z. Tan, Y. Kim, C. E. Kehayias, F. Streller, Y. R. Zhou, R. Carpick, Z. Luo, Y. W. Park, A. M. Rappe, M. Drndić, J. M. Kikkawa, A. T. C. Johnson, *2D Mater.* **2017**, *4*, 021008.

49 C. G. Kang, S. K. Lim, S. Lee, S. K. Lee, C. Cho, Y. G. Lee, H. J. Hwang, Y. Kim, H. J. Choi, S. H. Choe, M.-H. Ham, B. H. Lee, *Nanotechnology* **2013**, *24*, 115707.

50 E. Revolinsky, B. E. Brown, D. J. Beerntse C. H. Armitage, *J. Less-Common Met.* **1965**, *8*, 63.

51 F. Delogu, *Phys. Rev. B* **2005**, *72*, 205418.

52 Z. Chen, W. Jang, W. Bao, C. N. Lau, C. Dames, *Appl. Phys. Lett.* **2009**, *95*, 161910.

53 E. Yalon, C. J. McClellan, K. K. H. Smithe, M. M. Rojo, R. L. Xu, S. V. Suryavanshi, A. J. Gabourie, C. M. Neumann, F. Xiong, A. B. Farimani, E. Pop, *Nano Lett.* **2017**, *17*, 3429.

54 G. Liu, H. Y. Sun, J. Zhou, Q. F. Li, X.-G. Wan, *New J. Phys.* **2016**, *18*, 033017.

55 A. A. Vyas, C. Zhou, *IEEE Trans. on Nanotech.* **2018**, *17*, 4.

56 S. C. Sun, *Process technologies for advanced metallization and interconnect systems* in *Proc. Int. Electron Dev. Meeting Tech. Dig.*, IEEE, Washington DC, USA **1997**, pp. 765-768.

57 C. Adelmann, L. G. Wen, A. P. Peter, Y. K. Siew, K. Croes, J. Swerts, M. Popovici, K. Sannkaran, G. Pourtois, S. V. Elshocht, J. Bommels, Z. Tokei, *Alternative metals for advanced interconnects* in *Proc. IEEE Int. Interconnect Tech. Conf.*, IEEE, San Jose,

USA **2014**, pp. 173-176.

58 C. Zhou, A. A. Vyas, P. Wilhite, P. Wang, M. Chan, C. Y. Yang, *IEEE Electron Dev. Lett.* **2015**, *36*, 71.

59 T. Yu, C.-W. Liang, C. Kim, E.-S. Song, B. Yu, *IEEE Electron Dev. Lett.* **2011**, *32*, 1110.

60 Y. Yang, R. Murali., *IEEE Electron Dev. Lett.* **2010**, *31*, 237.
